# Effect of Empagliflozin on Heart Failure Outcomes After Acute Myocardial Infarction: Insights From the EMPACT-MI Trial

**DOI:** 10.1161/CIRCULATIONAHA.124.069217

**Published:** 2024-04-06

**Authors:** Adrian F. Hernandez, Jacob A. Udell, W. Schuyler Jones, Stefan D. Anker, Mark C. Petrie, Josephine Harrington, Michaela Mattheus, Svenja Seide, Isabella Zwiener, Offer Amir, M. Cecilia Bahit, Johann Bauersachs, Antoni Bayes-Genis, Yundai Chen, Vijay K. Chopra, Gemma A. Figtree, Junbo Ge, Shaun G. Goodman, Nina Gotcheva, Shinya Goto, Tomasz Gasior, Waheed Jamal, James L. Januzzi, Myung Ho Jeong, Yuri Lopatin, Renato D. Lopes, Béla Merkely, Puja B. Parikh, Alexander Parkhomenko, Piotr Ponikowski, Xavier Rossello, Morten Schou, Dragan Simic, Philippe Gabriel Steg, Joanna Szachniewicz, Peter van der Meer, Dragos Vinereanu, Shelley Zieroth, Martina Brueckmann, Mikhail Sumin, Deepak L. Bhatt, Javed Butler

**Affiliations:** Duke University Department of Medicine, Division of Cardiology, and Duke Clinical Research Institute, Durham, NC (A.F.H., W.S.J., J.H., R.D.L.).; Women’s College Hospital and Peter Munk Cardiac Centre, Toronto General Hospital, University of Toronto, Canada (J.A.U.).; Department of Cardiology (CVK) of German Heart Center Charité, Berlin Institute of Health Center for Regenerative Therapies (BCRT), German Centre for Cardiovascular Research (DZHK) partner site Berlin, Charité Universitätsmedizin, Berlin, Germany (S.D.A.).; School of Cardiovascular and Medical Sciences, British Heart Foundation Glasgow Cardiovascular Research Centre, University of Glasgow, UK (M.C.P.).; Boehringer Ingelheim Pharma GmbH & Co KG, Ingelheim, Germany (M.M., S.S., I.Z.).; Heart Institute, Hadassah Medical Center, The Hebrew University of Jerusalem, Israel (O.A.).; INECO Neurociencias Oroño, Fundación INECO, Rosario, Santa Fe, Argentina (M.C.B.).; Department of Cardiology and Angiology, Hannover Medical School, Germany (J. Bauersachs).; Heart Institute, Hospital Universitari Germans Trias i Pujol, Barcelona, Spain (A.B.-G.).; Department of Medicine, Universitat Autònomoa de Barcelona, Spain (A.B.-G.).; Department of Cardiology, the First Medical Center of Chinese PLA General Hospital, Beijing, China (Y.C.).; Max Super Speciality Hospital, Saket, New Delhi, India (V.K.C.).; Faculty of Medicine and Health, University of Sydney, Australia (G.A.F.).; Department of Cardiology, Zhongshan Hospital, Fudan University, Shanghai Institute of Cardiovascular Diseases, National Clinical Research Center for Interventional Medicine, Shanghai, China (J.G.).; Canadian VIGOUR Centre, University of Alberta, Edmonton, Canada (S.G.G.).; Division of Cardiology, Department of Medicine, St Michael’s Hospital, Unity Health Toronto and Peter Munk Cardiac Centre, University Health Network, University of Toronto, Canada (S.G.G.).; Department of Cardiology, MHAT “National Cardiology Hospital” EAD, Sofia, Bulgaria (N.G.).; Department of Medicine (Cardiology), Tokai University School of Medicine, Isehara, Japan (S.G.).; Boehringer Ingelheim International GmbH, Ingelheim, Germany (T.G., W.J., M.B., M. Sumin).; Collegium Medicum, Faculty of Medicine, WSB University, Dabrowa Gornicza, Poland (T.G.).; Division of Cardiology, Harvard Medical School and Massachusetts General Hospital, Boston (J.L.J.).; Chonnam National University Hospital and Medical School, Gwangju, Republic of Korea (M.H.J.).; Volgograd State Medical University, Russia (Y.L.).; Heart and Vascular Center, Semmelweis University, Budapest, Hungary (B.M.).; Division of Cardiovascular Medicine, Department of Medicine, State University of New York at Stony Brook (P.B.P.).; The Ukrainian Institute of Cardiology na MD Strazhesko, AMS Ukraine, Kyiv (A.P.).; Institute of Heart Diseases, Wroclaw Medical University, Poland (P.P.).; Hospital Universitari Son Espases, Health Research Institute of the Balearic Islands, University of the Balearic Islands, Palma de Mallorca, Spain (X.R.).; Department of Cardiology, Herlev and Gentofte University Hospital, Copenhagen, Denmark (M. Schou).; Department of Cardiovascular Diseases, University Clinical Center Belgrade, Serbia (D.S.).; Université Paris-Cité, FACT (French Alliance for Cardiovascular Trials), INSERM U-1148, AP-HP, Hôpital Bichat, Paris, France (P.G.S.).; Jan Mikulicz–Radecki University Clinical Hospital, Wroclaw, Poland (J.S.).; Department of Cardiology, University of Groningen, University Medical Centre Groningen, the Netherlands (P.v.d.M.).; University of Medicine and Pharmacy Carol Davila, University and Emergency Hospital, Bucharest, Romania (D.V.).; Section of Cardiology, Max Rady College of Medicine, University of Manitoba, Winnipeg, Canada (S.Z.).; First Department of Medicine, Faculty of Medicine Mannheim, University of Heidelberg, Mannheim, Germany (M.B.).; Mount Sinai Fuster Heart Hospital, Icahn School of Medicine at Mount Sinai, New York, NY (D.L.B.).; Baylor Scott and White Research Institute, Dallas, TX (J. Butler).; Department of Medicine, University of Mississippi, Jackson (J. Butler).

**Keywords:** heart failure, hospitalization, myocardial infarction

## Abstract

**BACKGROUND:**

Empagliflozin reduces the risk of heart failure (HF) events in patients with type 2 diabetes at high cardiovascular risk, chronic kidney disease, or prevalent HF irrespective of ejection fraction. Whereas the EMPACT-MI trial (Effect of Empagliflozin on Hospitalization for Heart Failure and Mortality in Patients With Acute Myocardial Infarction) showed that empagliflozin does not reduce the risk of the composite of hospitalization for HF and all-cause death, the effect of empagliflozin on first and recurrent HF events after myocardial infarction is unknown.

**METHODS:**

EMPACT-MI was a double-blind, randomized, placebo-controlled, event-driven trial that randomized 6522 patients hospitalized for acute myocardial infarction at risk for HF on the basis of newly developed left ventricular ejection fraction of <45% or signs or symptoms of congestion to receive empagliflozin 10 mg daily or placebo within 14 days of admission. In prespecified secondary analyses, treatment groups were analyzed for HF outcomes.

**RESULTS:**

Over a median follow-up of 17.9 months, the risk for first HF hospitalization and total HF hospitalizations was significantly lower in the empagliflozin compared with the placebo group (118 [3.6%] versus 153 [4.7%] patients with events; hazard ratio, 0.77 [95% CI, 0.60, 0.98]; *P*=0.031, for first HF hospitalization; 148 versus 207 events; rate ratio, 0.67 [95% CI, 0.51, 0.89]; *P*=0.006, for total HF hospitalizations). Subgroup analysis showed consistency of empagliflozin benefit across clinically relevant patient subgroups for first and total HF hospitalizations. The need for new use of diuretics, renin-angiotensin modulators, or mineralocorticoid receptor antagonists after discharge was less in patients randomized to empagliflozin versus placebo (all *P*<0.05).

**CONCLUSIONS:**

Empagliflozin reduced the risk of HF in patients with left ventricular dysfunction or congestion after acute myocardial infarction.

**REGISTRATION:**

URL: https://www.clinicaltrials.gov; Unique identifier: NCT04509674.

**Clinical Perspective**

**What Is New?**

- Empagliflozin reduced the risk of first and total heart failure (HF) hospitalizations by 23% and 33%, respectively, in patients with left ventricular dysfunction or congestion after acute myocardial infarction.
- The benefit of empagliflozin was consistent across various patient subgroups and across a broad range of HF outcomes, including adverse events of HF and initiation of HF therapies after discharge.

**What Are the Clinical Implications?**

- Acute myocardial infarction is frequently complicated by new onset of HF or leads to the development of chronic HF after discharge.
- Empagliflozin may have a role for the prevention of HF in high-risk patients after myocardial infarction, especially those with left ventricular dysfunction or congestion.

Acute myocardial infarction (MI) is frequently complicated by new onset of heart failure (HF) or leads to the development of chronic HF after discharge.^1^ Once HF manifests after MI, it is associated with higher death and recurrent hospitalization risk, as well as other complications.^2^ Guidelines emphasize the identification of high-risk patients and the implementation of therapeutic interventions to prevent the development and progression of HF.^3,4^ Sodium-glucose cotransporter-2 (SGLT2) inhibitors have been consistently shown to reduce the risk of HF events in patients at high risk of developing HF (eg, those with type 2 diabetes at high cardiovascular risk or chronic kidney disease) as well as those with prevalent HF, irrespective of left ventricular ejection fraction (LVEF).

Empagliflozin has been shown to reduce the risk markers associated with developing HF after an acute MI, including lowering natriuretic peptide levels, improving LVEF, and decreasing cardiac volumes.^5^ In the DAPA-MI trial (Dapagliflozin Effects on Cardiometabolic Outcomes in Patients With an Acute Heart Attack), results for HF outcomes with dapagliflozin were inconclusive, as the primary end point was changed to a 7-level win ratio because of the lower than expected event rate.^6^

The EMPACT-MI trial (Effect of Empagliflozin on Hospitalization for Heart Failure and Mortality in Patients With Acute Myocardial Infarction) was designed to assess clinical outcomes after MI. The primary results have recently been reported.^7^ Although empagliflozin did not reduce the primary composite end point of time to first hospitalization for HF or all-cause death (hazard ratio [HR], 0.90; 95% CI, 0.76–1.06; *P*=0.21), the effect of empagliflozin on HF outcomes after MI remains clinically important.^8^ In this report, we provide detailed data on prespecified analyses of HF hospitalizations and other analyses related to HF events after acute MI at risk for development of HF with either newly developed LVEF of <45% or signs or symptoms of congestion requiring treatment.

## METHODS

### Trial Design

EMPACT-MI was an international, randomized, parallel-group, double-blind, placebo-controlled, event-driven trial. The trial design and primary results have been described previously.^7^ The trial protocol was developed and amended by the executive and steering committees. The executive committee provided scientific oversight of the development of the statistical analysis plan, patient recruitment and follow-up, and data analysis, including prespecified secondary analyses on subsequent development of HF. An independent data monitoring committee reviewed the safety data. The trial was approved by the ethics committee at each trial site and all patients provided written informed consent. Statistical analyses for this analysis were performed by employees of the sponsor under the oversight of the executive committee.

### Patient Population

EMPACT-MI randomized patients 18 years of age or older hospitalized with an acute MI within 14 days of admission. Patients with ST-segment–elevation MI (STEMI) or non-STEMI were eligible. Patients with or without type 2 diabetes were included. Before randomization, the patients had either evidence of newly developed LEVF<45% or signs (eg, pulmonary rales, crackles, or crepitations; elevated jugular venous pressure; congestion on chest x-ray) or symptoms (eg, dyspnea, decreased exercise tolerance, or fatigue) of congestion requiring treatment (eg, augmentation or initiation of oral diuretic therapy, intravenous diuretic therapy, intravenous vasoactive agent, mechanical intervention). Patients were required to have at least 1 enrichment factor, including ≥65 years of age, newly developed LEVF<35%, history of MI, atrial fibrillation, type 2 diabetes, estimated glomerular filtration rate <60 mL/min/1.73 m^2^, elevated natriuretic peptides or uric acid levels, elevated pulmonary artery or right ventricular systolic pressure, 3-vessel coronary artery disease, peripheral artery disease, or no revascularization for index MI. Patients with a previous diagnosis of HF or who were taking or planned the use of SGLT2 inhibitors were excluded. Further details of the study population, including the baseline characteristics and a full list of eligibility criteria, have been previously reported, and are listed in the Supplemental Material.^9^

### Study Procedures

Patients were randomized to either placebo or 10 mg of empagliflozin daily in a 1:1 ratio, in addition to standard of care. In this streamlined trial, after randomization, participants had a remote visit at 2 weeks, followed by a face-to-face visit at 6 months after randomization. Patients continued to have remote visits every 6 months until end of study, when a final telephone call visit was performed. During these visits, information on prespecified end points, safety events, and adherence to study drug was collected. Data on any concomitant medications were collected for 6 months after randomization, except for the initiation of nonstudy open-label SGLT2 or SGLT1/2 inhibitor, which was collected throughout the trial. All randomized patients were followed for the duration of the trial, regardless of intake of study medications.

### Study Outcomes

The primary end point for EMPACT-MI was the composite of time to first hospitalization for HF or all-cause death and has been previously reported.^8^ The key secondary outcomes were total number of hospitalizations for HF or all-cause death, total number of nonelective cardiovascular hospitalizations or all-cause death, total number of nonelective all-cause hospitalizations or all-cause death, and total number of hospitalizations for MI or all-cause death, and have also been previously reported.^8^

### HF Outcomes (Including Hospitalization With HF as the Primary Reason)

For this report, the focus is on HF outcomes as prespecified from the protocol as described previously and from a prespecified publication analytical plan for HF-related events. These additional HF-related end points studied in this investigation included prespecified time to first HF hospitalization and prespecified total (first and recurrent) HF hospitalizations. We also investigated time to first HF hospitalization or death attributable to HF and total HF hospitalizations or death attributable to HF in exploratory post hoc analyses.

### HF Adverse Events

In exploratory analyses, we also examined investigator-reported adverse events (AEs) that were categorized as cardiac failure per MedDRA standards and included not only the events analyzed as the prespecified end point of HF hospitalization but a broader range of AEs of HF, including outpatient nonfatal AEs as well as those requiring or prolonging hospitalization or with a fatal outcome. For a full list of all preferred terms and details of the methodology of the evaluation of AEs of HF, refer to the Appendix in the Supplemental Material.

For these exploratory analyses, we examined time to first AE and total number of AEs of HF, time to first AE and total number of AEs of HF requiring or prolonging hospitalization or with fatal outcome, time to first AE and total number of outpatient nonfatal AEs of HF, as well as time to first event and total number of AEs of HF or all-cause death and HF or cardiovascular death.

### End Point Ascertainment

End points of HF hospitalization and HF death were assessed by investigators blinded to study drug assignment who received standardized training and were monitored for quality assurance. Investigators were trained to report events on the basis of prespecified definitions consistent with previous guidance on cardiovascular event classification. These end points were verified according to the prespecified algorithm as previously described and shown in the Supplemental Material for outcome events definitions.

### Statistical Analysis

The analyses were performed on the basis of the intention-to-treat principle and included all randomized study patients. Comparison between the empagliflozin and placebo arms for time to first event end points was performed using primary Cox proportional hazards model, including baseline covariates of age, geographic region, estimated glomerular filtration rate (assessed categorically using the CKD-EPI [Chronic Kidney Disease Epidemiology Collaboration] formula <45 versus 45 to <60 versus 60 to <90 versus ≥90 mL/min/1.73 m^2^), LEVF(<35% versus ≥35%), type 2 diabetes, persistent or permanent atrial fibrillation, previous MI, peripheral artery disease, and smoking status. Data for patients who did not have an event were censored on the last day they were known to be free of the outcome. The assumption of proportional hazards was verified. For comparison of total (first and recurrent) events, differences between empagliflozin and placebo were assessed using a negative binomial model including the same covariates that were used for time to first event analyses and including logarithm of time as an adjustment for observation time. Sensitivity analyses were performed to assess the robustness of the results for time to first HF hospitalization and for total HF hospitalizations, including analyses on the basis of inclusion of a broader spectrum of HF hospitalizations that were not included in the primary analysis (as described in Study Outcomes and the Supplemental Material) and analyses on the basis of models including only stratification variables of type 2 diabetes and geographic region.

Total hospitalizations for HF events were further assessed in a time-to-event analysis using the prespecified Wei-Lin-Weissfeld model,^11^ which produces estimated relative treatment effects in terms of the HR for the individual first and recurrent events by the order in which they occur (HR for first event, HR for second event, and HR for third event). This model also includes a test of the consistency of the treatment estimates across the individual order of sequential events.

To assess the possible effect of death as a competing risk, we performed sensitivity analyses using the prespecified semiparametric joint frailty model with a piecewise constant Weibull baseline hazard for total number of hospitalizations for HF considering all-cause death as competing risk.^10^

Consistency of the effect of empagliflozin on time to first HF hospitalization and total HF hospitalizations were assessed across a broad range of prespecified patient subgroups, including age (<65 or ≥65 years), sex, region, ethnicity, race, type of index MI (STEMI or non-STEMI), type 2 diabetes, baseline estimated glomerular filtration rate (≥60 or <60 mL/min/1.73 m^2^), systolic blood pressure (<110, ≥110 to 130, or ≥130 mm Hg), history of MI, persistent or permanent atrial fibrillation, median time from index MI diagnosis to randomization, and treatment with angiotensin receptor-neprilysin inhibitor (ARNI), angiotensin-converting enzyme inhibitor, angiotensin receptor blocker, β-blocker, mineralocorticoid receptor antagonist (MRA), or loop/high-ceiling diuretic. These analyses were performed on the basis of the Cox regression and negative binomial regression models including factors as described previously and additional terms for subgroup and interaction of subgroup by treatment (with interaction tests for categorical variables and trend tests for ordered variables).

An analysis was performed to assess new initiation of HF medications after discharge until 6 months after randomization for time to first initiation of diuretics (other than MRAs), ARNI, angiotensin-converting enzyme inhibitor, angiotensin receptor blocker or ARNI, MRA, and beta-blockers in patients not on these respective medication at discharge. Time to first implantation of either implantable cardiac defibrillator or cardiac resynchronization therapy device was also assessed.

All *P* values reported for these exploratory analyses are 2-sided, and *P*<0.05 was considered statistically significant.

To ensure independent interpretation of clinical study results and enable authors to fulfil their role and obligations under the International Committee of Medical Journal Editors criteria, Boehringer Ingelheim grants all external authors access to relevant clinical study data. In adherence with the Boehringer Ingelheim Policy on Transparency and Publication of Clinical Study Data, scientific and medical researchers can request access to clinical study data typically 1 year after the approval has been granted by major regulatory authorities or after termination of the development program. Researchers should use https://vivli.org to request access to study or visit data.

## RESULTS

### Baseline Characteristics

From December 2020 through March 2023, 6522 patients were randomly assigned to receive empagliflozin (n=3260) or placebo (n=3262) at 451 sites in 22 countries. Baseline characteristics were balanced between study drug groups (Table S2). The randomized population had an LVEF <45% in 78.4% of patients and 57.0% had signs or symptoms of congestion that required treatment during index hospitalization. The most common enrichment factors included age ≥65 years (50.0%), type 2 diabetes (31.9%), and 3-vessel coronary disease (31.0%). A total of 6328 (97.0%) patients were followed for primary end point until trial end and 6467 (99.2%) provided vital status at the end of the trial.^8,9^ Median follow-up duration was 17.9 months and median drug exposure was comparable between the 2 arms.^8^

### HF Outcomes (Including Hospitalization With HF as the Primary Reason)

**Figure 1.**
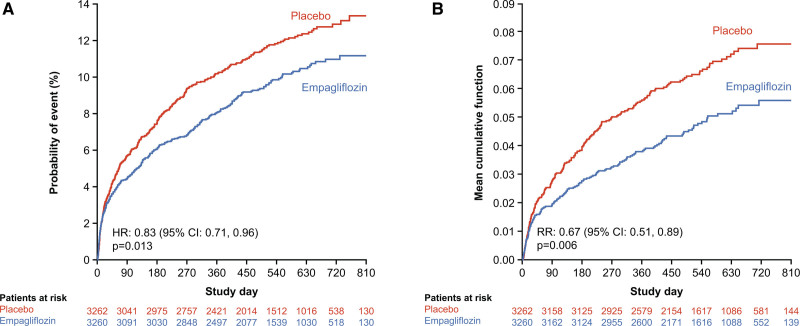
**Time to first adverse event of heart failure or all-cause death and total number of heart failure hospitalizations. A**, Time to first adverse event of heart failure or all-cause death. **B**, Total number of heart failure hospitalizations. HR indicates hazard ratio; and RR, rate ratio.

**Figure 2.**
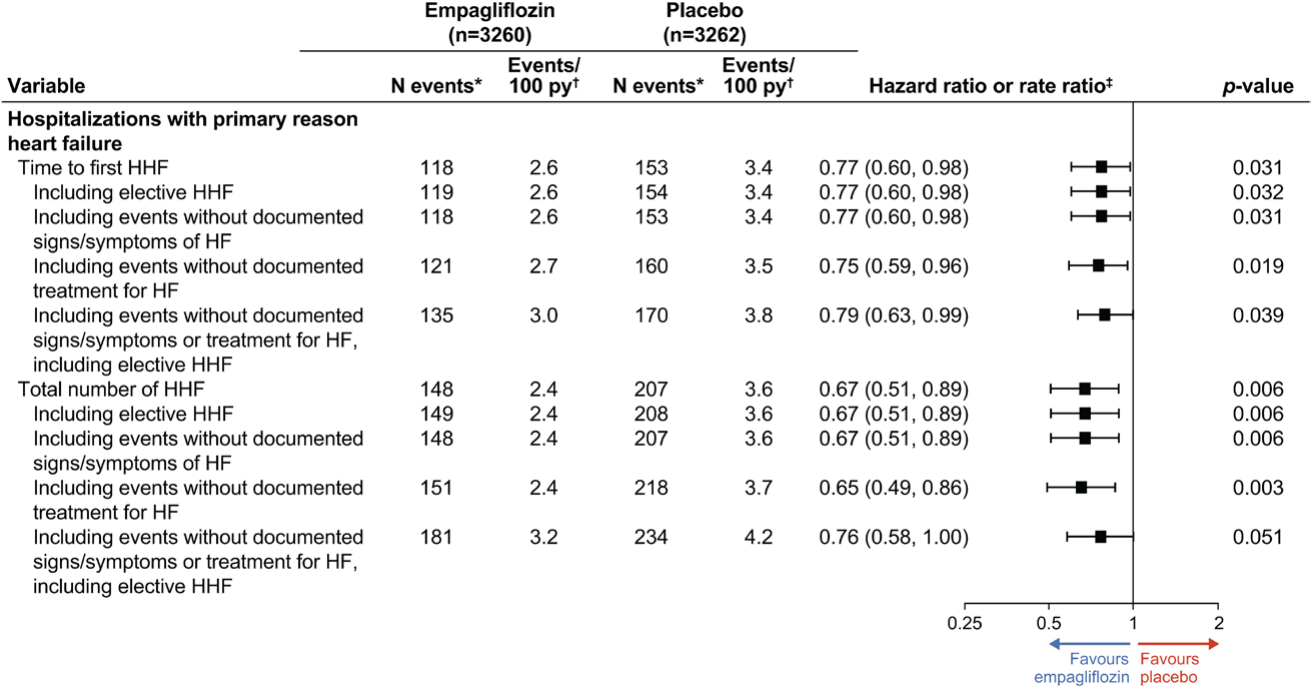
**Major heart failure outcomes.** *Number of patients with events for time to first event end points and number of events for total number of events end points. †Number of patients with events per 100 patient-years for time to first event end points and adjusted number of events per 100 patient-years (on the basis of negative binomial regression) for total number of event end points. ‡Hazard ratio (95% CI), *P* value on the basis of Cox proportional hazards model for time to first event end points, event rate ratio (95% CI) on the basis of negative binomial regression for total number of events end points. HHF indicates hospitalization for heart failure.

The risks of first HF hospitalization and the total number of HF hospitalizations were significantly reduced with empagliflozin compared with placebo (118 versus 153 events; HR, 0.77 [95% CI, 0.60, 0.98]; *P*=0.031 for first HF hospitalization and 148 versus 207 events; rate ratio [RR], 0.67 [95% CI, 0.51, 0.89]; *P*=0.006 for total HF hospitalizations; Figure 1B; Figure 2; Figure S3).

**Figure 3.**
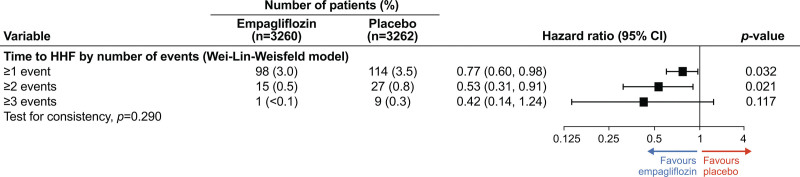
**Time-to-event analyses of hospitalization for heart failure by order of event according to the Wei-Lin-Weissfeld model.** HHF indicates hospitalization for heart failure.

Overall, 271 (4.16%) patients had at least one HF hospitalization, and 59 (0.9%) subsequently had a recurrent event, with a total of 355 HF hospitalization events. Analyses of total events by the order of events on the basis of the Wei-Lin-Weissfeld model (ie, time to first event, time to second event, and time to third event) showed a consistent effect of empagliflozin (test for consistency of the effect across the order of events *P*=0.29; Figure 3).The sensitivity analyses for total number of HF hospitalizations using a joint frailty model to account for the competing risk of death gave consistent results compared with the results using the negative binomial model (Figure S2).

In exploratory analyses, the risks of first HF hospitalization or death attributable to HF (129 versus 166 events; HR, 0.78 [95% CI, 0.62, 0.98]; *P*=0.031) and total HF hospitalizations or death attributable to HF (168 versus 236 events; RR, 0.69 [95% CI, 0.51, 0.93]; *P*=0.015) were significantly reduced in the empagliflozin compared with the placebo arm (Figures S3 and S4).

The sensitivity analyses of time to first and total HF hospitalizations including broader definitions of HF hospitalization showed consistent risk reductions with empagliflozin with those from the main analyses (Figure 2).

First and total HF hospitalization sensitivity analyses on the basis of a model including only the stratification factors provided consistent results with those from the main analyses (Figure S2). About half of the first HF hospitalizations appeared within the first 89 days after randomization and fewer patients on empagliflozin were hospitalized for HF within the first 89 days (56 [1.7%] versus 77 [2.4%]) and after 89 days (62 [2.0%] versus 76 [2.5%]) from randomization.

### HF Adverse Events

To assess the totality of HF events reported in the trial, we evaluated AEs of HF, which included not only events analyzed as prespecified end point of HF hospitalization but a broader spectrum of AEs including those requiring or prolonging hospitalization or with fatal outcome as well as outpatient nonfatal events. When analyzing total number of adverse HF events requiring or prolonging hospitalization or with fatal outcome, there were 497 events in total, and for total AEs of HF that include outpatient events, there were in total 581 events. When assessing AEs of HF requiring or prolonging hospitalization or with fatal outcome, there was a significantly lower risk of experiencing a first event (HR, 0.78 [95% CI, 0.63, 0.96]; *P*=0.02; Figures S5 and S6) and a lower risk in the total number of events (RR, 0.66 [95% CI, 0.50, 0.87]; *P*=0.0035; Figures S5 and S6) in the empagliflozin versus placebo group. For outpatient nonfatal AEs of HF, there was a significantly lower risk of time to first event (HR, 0.48 [95% CI, 0.31, 0.73]; *P*=0.0005; Figures S5 and S6) and total number of events (RR, 0.51 [95% CI, 0.33, 0.80]; *P*=0.0035; Figures S5 and S6). In addition, for the AEs of HF, there was a significantly lower risk of time to first event (HR, 0.70 [95% CI, 0.57, 0.84]; *P*=0.0002; Figures S4 through S6) and total number of events (RR, 0.63 [95% CI, 0.50, 0.79]; *P*<0.0001; Figures S5 and S6) with empagliflozin versus placebo. Significantly lower risk with empagliflozin versus placebo was also shown in the analysis of the composite end point of time to first AE of HF or all-cause mortality (HR, 0.83 [95% CI, 0.71, 0.96]; *P*=0.013 on the basis of 690 events; Figure 1A; Figure S6) and total number of AEs of HF or all-cause death (RR, 0.79 [95% CI, 0.63, 0.98]; *P*=0.031 on the basis of 928 events; Figure S6) as well as time to first AE of HF or cardiovascular death (HR, 0.82 [95% CI, 0.70, 0.96]; *P*=0.013; Figure S6) and total number of AEs of HF or cardiovascular death (RR, 0.79 [95% CI, 0.63, 0.99]; *P*=0.043; Figure S6).

### Subgroup Analysis

**Figure 4.**
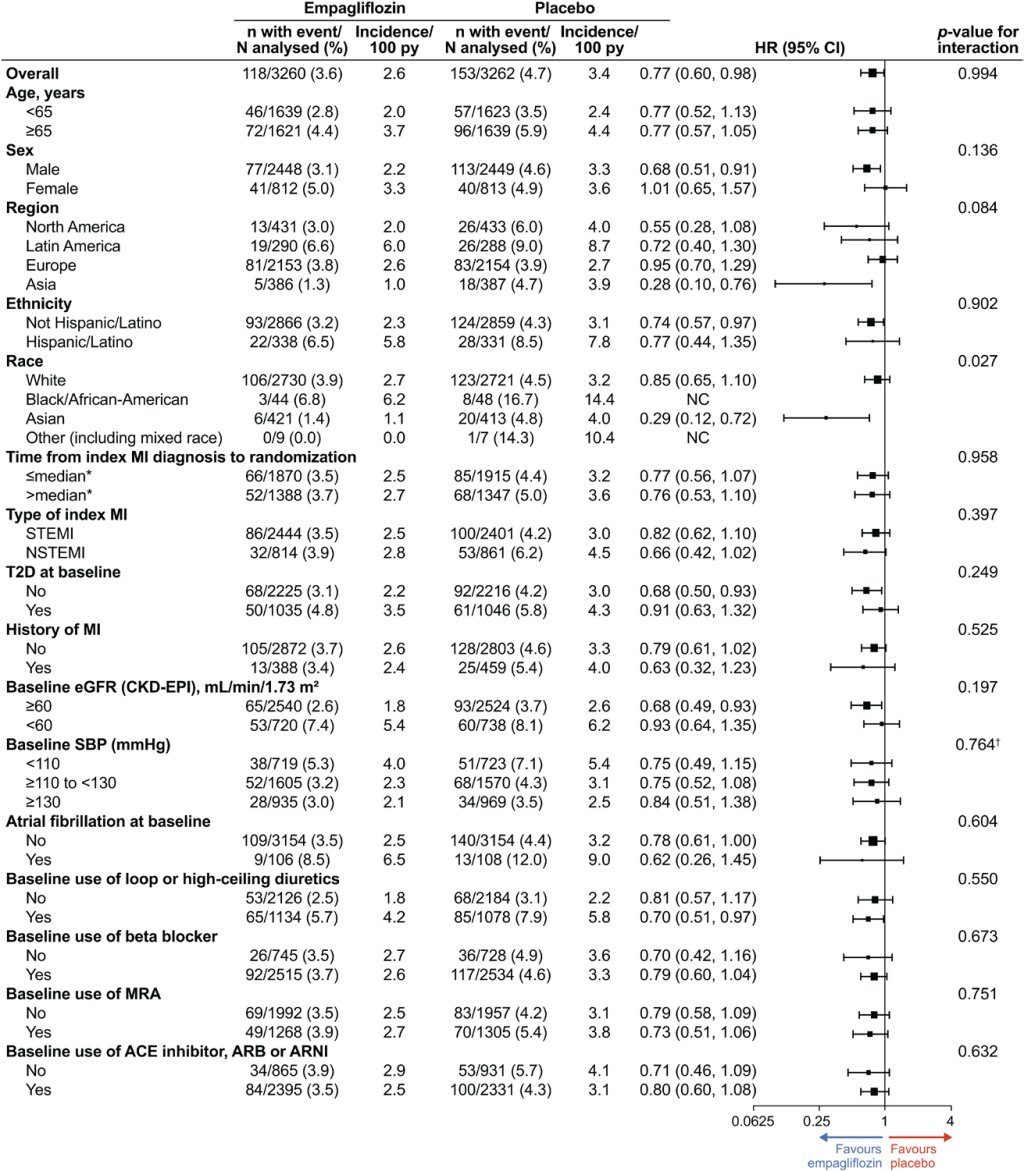
**Time to first heart failure hospitalization, according to prespecified subgroups.** *Median time from index myocardial infarction (MI) diagnosis to randomization: 5.0 days. ACE indicates angiotensin-converting enzyme; ARB, angiotensin receptor blocker; ARNI, angiotensin receptor-neprilysin inhibitor; CKD-EPI, Chronic Kidney Disease Epidemiology Collaboration; eGFR, estimated glomerular filtration rate; HR, hazard ratio; MRA, mineralocorticoid receptor antagonist; NC, not calculated; NSTEMI, non–ST-segment–elevation myocardial infarction; SBP, systolic blood pressure; STEMI, ST-segment–elevation myocardial infarction; and T2D, type 2 diabetes.

**Figure 5.**
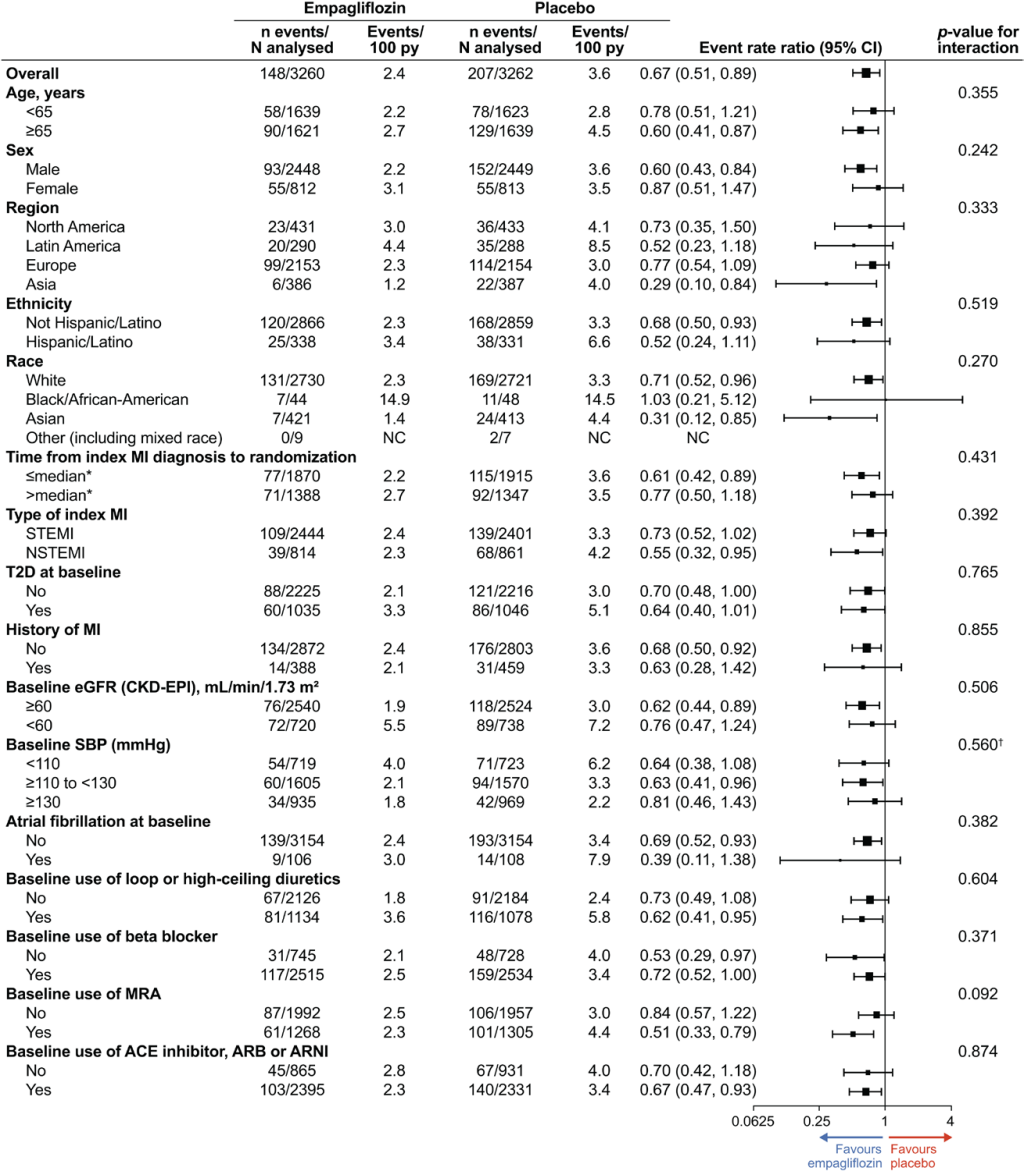
**Total number of heart failure hospitalizations, according to prespecified subgroups.** *Median time from index myocardial infarction (MI) diagnosis to randomization: 5.0 days. ACE indicates angiotensin-converting enzyme; ARB, angiotensin receptor blocker; ARNI, angiotensin receptor-neprilysin inhibitor; CKD-EPI, Chronic Kidney Disease Epidemiology Collaboration; eGFR, estimated glomerular filtration rate; MRA, mineralocorticoid receptor antagonist; NC, not calculated; NSTEMI, non–ST-segment–elevation myocardial infarction; SBP, systolic blood pressure; STEMI, ST-segment–elevation myocardial infarction; and T2D, type 2 diabetes.

The risk reduction with empagliflozin versus placebo was generally consistent across patient baseline subgroups studied, including clinically relevant subgroups by age, sex, region, race, ethnicity, persistent or permanent atrial fibrillation, type of index MI (STEMI versus non-STEMI), type 2 diabetes (yes versus no), baseline estimated glomerular filtration rate (≥60 versus <60 mL/min/1.73 m^2^), or treatment with angiotensin-converting enzyme inhibitor/angiotensin receptor blocker/ARNI, β-blocker, MRA, and diuretics for both time to first HF hospitalization (Figure 4) and total (first and recurrent) HF hospitalizations (Figure 5). The only statistically positive interaction was observed for time to first HF hospitalization for race (*P*=0.027); although the HRs were <1 for all categories, there were very few events among non-White patients.

### HF Therapy After Discharge

**Figure 6.**
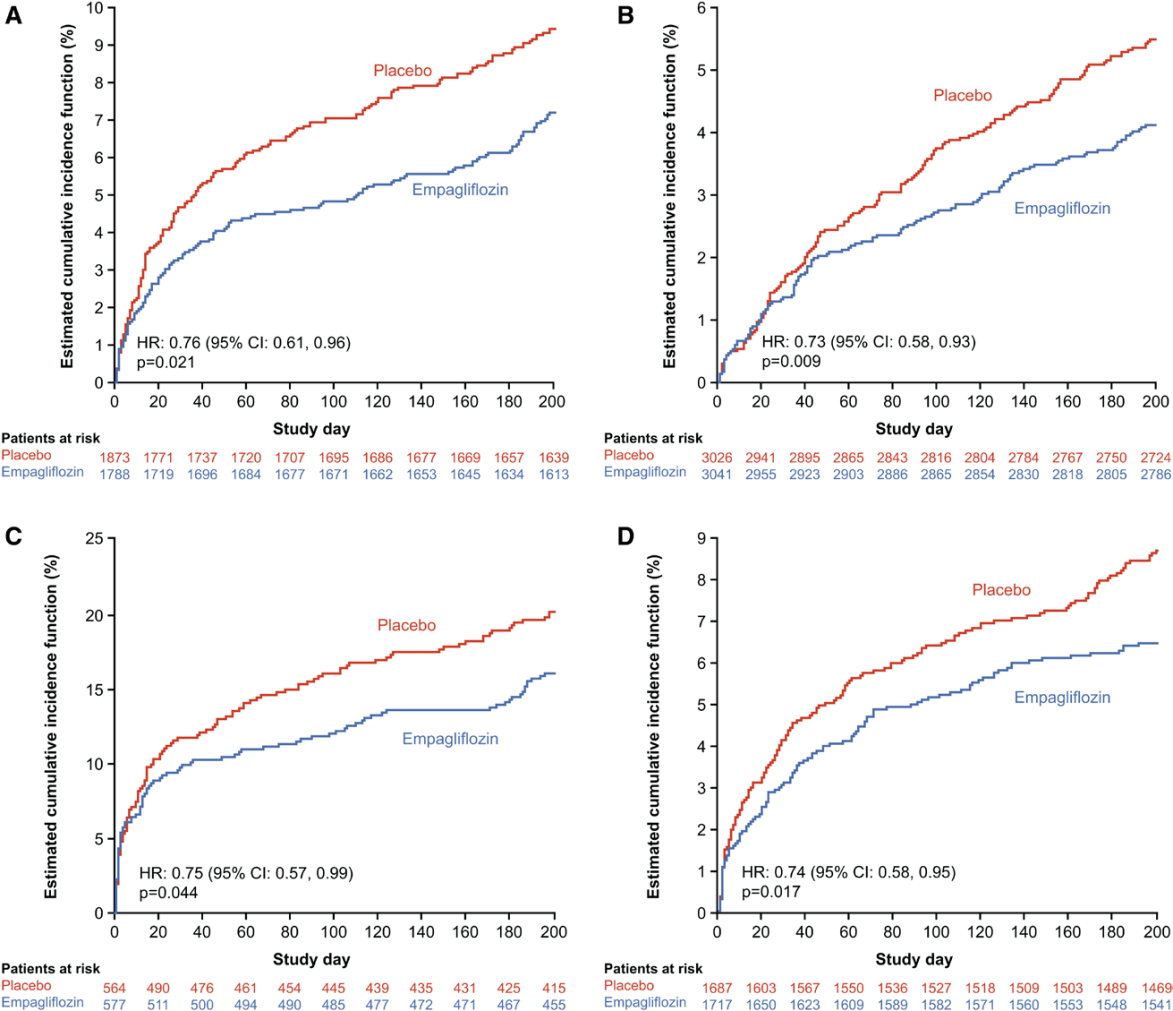
**Cumulative incidence function for time to first use of heart failure therapies after discharge until 6 months. A**, Diuretics (excluding mineralocorticoid receptor antagonist). **B**, Angiotensin receptor-neprilysin inhibitor. **C**, Angiotensin-converting enzyme inhibitor, angiotensin receptor blocker, or angiotensin receptor-neprilysin inhibitor. **D**, Mineralocorticoid receptor antagonist. HR indicates hazard ratio.

Among patients discharged without diuretic therapy, significantly fewer patients in the empagliflozin arm were started on a diuretic other than MRA within 6 months after discharge compared with those randomized to placebo (n=138 [12.2%] versus n=174 [15.3%]; HR, 0.80 [95% CI, 0.64, 1.00]; *P*=0.046; Figure 6A). Significantly fewer patients were initiated on ARNI (HR, 0.73 [95% CI, 0.58, 0.93]; *P*=0.009; Figure 6B); angiotensin-converting enzyme inhibitor, angiotensin receptor blocker, or ARNI (HR, 0.75 [95% CI, 0.57, 0.99]; *P*=0.044; Figure 6C); or MRA (HR, 0.74 [95% CI, 0.58, 0.95]; *P*=0.017; Figure 6D) among patients not on these therapies at discharge. There was lower initiation of beta-blockers (HR, 0.75 [95% CI, 0.55, 1.04]; *P*=0.084) and implantation of implantable cardiac defibrillator or cardiac resynchronization therapy in empagliflozin versus the placebo group (n=68 [2.1%] versus n=85 [2.6%]; HR, 0.80 [95% CI, 0.58, 1.10]; *P*=0.16).

## DISCUSSION

Whereas empagliflozin did not reduce the primary composite end point of hospitalization for HF or all-cause death in the EMPACT-MI trial, as previously reported,^8^ we show in these prespecified analyses that empagliflozin reduced the time to first hospitalization for HF and the total number of hospitalizations for HF. These benefits were consistent across relevant patient subgroups, including those with STEMI or non-STEMI and those with or without type 2 diabetes. We also observed consistent benefits of empagliflozin in exploratory analyses in the risk reduction of HF AEs, including the full range of HF events such as prolonged hospitalization because of HF or outpatient HF events. These results are consistent with previous trials with SGLT2 inhibitors in other patient populations and highlight the role of empagliflozin in preventing HF after MI.^11^

Clinical guidelines emphasize the early detection of patients at risk for developing HF.^3,4^ Acute MI complicated by symptoms of HF or left ventricular dysfunction underscores a high risk and portends poor outcomes.^1^ If patients do not recover left ventricular function after an ischemic event, they are particularly vulnerable for developing chronic HF and subsequent high risk for death and hospitalizations. With these results of EMPACT-MI, we show that empagliflozin has a role for the treatment of patients after acute MI who are at risk for HF by reducing the burden of development of HF. Empagliflozin reduced the risk of time to first hospitalization for HF by 23% and the total number of hospitalizations for HF by 33%. These results are not only directionally but quantitatively comparable to the effect of SGLT2 inhibitors seen in other patient populations, with benefits seen early, as has been described previously.^11^

Other studies have aimed to lower the long-term risk in patients with acute MI complicated by HF symptoms or left ventricular dysfunction. In PARADISE-MI (Prospective ARNI versus ACE Inhibitor Trial to Determine Superiority in Reducing Heart Failure Events after MI), valsartan-sacubitril did not lower the rate of death from cardiovascular causes or incident of HF compared with ramipril.^12^ There was also no reduction in the secondary end point of the risk of composite of hospitalization for HF or outpatient HF visits (HR, 0.84 [95% CI, 0.70, 1.02]). Whereas DAPA-MI showed that dapagliflozin improved cardiometabolic measures such as weight loss and new onset of diabetes, there was no significant improvement observed in the composite of cardiovascular death or hospitalization for HF compared with placebo (HR, 0.95 [95% CI, 0.64, 1.40]).^6^ However, the number of events for this composite was only 102, making the trial underpowered to assess these clinical outcomes reliably.

As the totality of evidence for SGLT2 inhibitors has evolved, it has become clear that HF benefits extend to patient populations with and without diabetes, as well as with and without previous HF, and across the spectrum of LVEF. We also found that empagliflozin reduces the risk of HF across important subgroups, including older patients, those with and without type 2 diabetes, STEMI or non-STEMI, and irrespective of background medical therapies, providing evidence for generalizable benefit in high-risk patients.

EMPACT-MI was conducted during the COVID-19 pandemic and previous studies have shown that the number of hospitalizations for HF substantially decreased during this period. Patients with tolerable symptoms may not have sought care or may have been managed in the outpatient setting, as observed through AE reporting. Moreover, 2 regions where the trial was conducted were affected by war during the trial. Also, being a streamlined trial by design that did not include a central event adjudication committee, only HF hospitalization events were considered for primary analyses. Recent trials have reported that outpatient HF events contribute meaningfully to the total HF burden; for example, of the 4744 patients in DAPA-HF (Dapagliflozin and Prevention of Adverse Outcomes in Heart Failure), there were a total of 549 hospitalizations for HF and 604 outpatient worsening of HF events.^13^ Indeed, we captured AEs related to HF because they were included in a prespecified list of AEs that were to be always reported as serious. When considering all these reported AEs of HF (including with fatal outcome, requiring or prolonging hospitalization, and outpatient events), the number of events analyzed was 581 in EMPACT-MI.

An indirect measure to assess new-onset HF burden after MI may be the initiation of typical HF therapies after discharge. Considering the streamlined nature of the study, medication data were collected only within the first 6 months after randomization (except for open-label SGLT2 or SGLT1/2 inhibitor use data, which were collected throughout the trial). Even with this limitation, there was a significantly lower rate of patients starting diuretics (other than MRA), ARNI, RAAS inhibitors, or MRA after discharge, further highlighting the effect of empagliflozin on clinical decisions requiring escalation of other medical therapy.

By design, this trial focused on clinical outcomes meaningful to clinicians and did not collect mechanistic data that would fully explain any results. The trial focused on randomizing patients early after MI, a dynamic time in which patients can have stunned myocardium that may recover especially after revascularization and independent of concomitant pharmacotherapy. The outcomes of HF hospitalizations were assessed by trained site investigators according to prespecified definitions with collection of corresponding data in structured electronic case report forms as described in the Supplemental Material and evaluated through monitoring for completeness. Therefore, end points were not centrally adjudicated. A limitation of these analyses of HF end points is that the primary end point of the trial was not met, so in a strict statistical sense should be considered exploratory. However, the reduction in HF outcomes observed is internally consistent within the trial across several definitions and subgroups as well as externally consistent with multiple other SGLT2 inhibitor trials in various clinical settings.

Whereas empagliflozin was not associated with a reduction in the risk of death after MI, the risk related to HF hospitalization was statistically significantly lower in patients randomized to empagliflozin compared with placebo. The magnitude of benefit observed was similar to that in previous trials involving SGLT2 inhibitors. Consistent benefit was seen across major patient subgroups as well as over the entire observation period, starting early after the index MI and across a broad range of HF outcomes. These data suggest the potential role for empagliflozin in preventing HF hospitalizations for high-risk patients after MI.

## ARTICLE INFORMATION

### Sources of Funding

Funding was provided by Boehringer Ingelheim and Eli Lilly and Company.

### Disclosures

Dr Hernandez has served as a consultant for Amgen, AstraZeneca, Bayer, Boehringer Ingelheim, Boston Scientific, Bristol Myers Squibb, Cytokinetics, Eidos, GlaxoSmithKline, Intellia, Intercept, MyoKardia, Novartis, Novo Nordisk, Prolaio, and TikkunLev; and has received research funding from American Regent, Amgen, Bayer, Boehringer Ingelheim, Lilly, Merck, Novartis, Novo Nordisk, and Verily. Dr Bhatt has served on advisory boards for ANGIOWave, Bayer, Boehringer Ingelheim, CellProthera, Cereno Scientific, Elsevier Practice Update Cardiology, High Enroll, Janssen, Level Ex, McKinsey, Medscape Cardiology, Merck, MyoKardia, NirvaMed, Novo Nordisk, PhaseBio, PLx Pharma, Stasys; on the board of directors for American Heart Association New York City, ANGIOWave (stock options), Bristol Myers Squibb (stock), DRS.LINQ (stock options), and High Enroll (stock); has served as a consultant for Broadview Ventures, GlaxoSmithKline, Hims, SFJ, and Youngene; has served on data monitoring committees for Acesion Pharma, Assistance Publique-Hôpitaux de Paris, Baim Institute for Clinical Research (formerly Harvard Clinical Research Institute, for the PORTICO trial, funded by St Jude Medical, now Abbott), Boston Scientific (chair, PEITHO trial), Cleveland Clinic, Contego Medical (chair, PERFORMANCE 2), Duke Clinical Research Institute, Mayo Clinic, Mount Sinai School of Medicine (for the ENVISAGE trial, funded by Daiichi Sankyo; for the ABILITY-DM trial, funded by Concept Medical; for ALLAY-HF, funded by Alleviant Medical), Novartis, Population Health Research Institute, and Rutgers University (for the NIH-funded MINT Trial); has received honoraria from American College of Cardiology (senior associate editor, Clinical Trials and News, ACC.org; chair, ACC accreditation oversight committee), Arnold and Porter law firm (work related to Sanofi/Bristol Myers Squibb clopidogrel litigation), Baim Institute for Clinical Research (formerly Harvard Clinical Research Institute; RE-DUAL PCI clinical trial steering committee funded by Boehringer Ingelheim; AEGIS-II executive committee funded by CSL Behring), Belvoir Publications (editor-in-chief, *Harvard Heart Letter*), Canadian Medical and Surgical Knowledge Translation Research Group (clinical trial steering committees), CSL Behring (AHA lecture), Cowen and Company, Duke Clinical Research Institute (clinical trial steering committees, including for the PRONOUNCE trial, funded by Ferring Pharmaceuticals), HMP Global (editor-in-chief, *Journal of Invasive Cardiology*), *Journal of the American College of Cardiology* (Guest editor; associate editor), K2P (co-chair, interdisciplinary curriculum), Level Ex, Medtelligence/ReachMD (CME steering committees), MJH Life Sciences, Oakstone CME (course director, Comprehensive Review of Interventional Cardiology), Piper Sandler, Population Health Research Institute (for the COMPASS operations committee, publications committee, steering committee, and USA national co-leader, funded by Bayer), WebMD (CME steering committees), and Wiley (steering committee); other: *Clinical Cardiology* (deputy editor); is named on a patent for sotagliflozin assigned to Brigham and Women’s Hospital, which was assigned to Lexicon (neither Dr Bhatt nor Brigham and Women’s Hospital receives any income from this patent); has received research funding from Abbott, Acesion Pharma, Afimmune, Aker Biomarine, Alnylam, Amarin, Amgen, AstraZeneca, Bayer, Beren, Boehringer Ingelheim, Boston Scientific, Bristol Myers Squibb, Cardax, CellProthera, Cereno Scientific, Chiesi, CinCor, Cleerly, CSL Behring, Eisai, Ethicon, Faraday Pharmaceuticals, Ferring Pharmaceuticals, Forest Laboratories, Fractyl, Garmin, HLS Therapeutics, Idorsia, Ironwood, Ischemix, Janssen, Javelin, Lexicon, Lilly, Medtronic, Merck, Moderna, MyoKardia, NirvaMed, Novartis, Novo Nordisk, Otsuka, Owkin, Pfizer, PhaseBio, PLx Pharma, Recardio, Regeneron, Reid Hoffman Foundation, Roche, Sanofi, Stasys, Synaptic, The Medicines Company, Youngene, and 89Bio; has received royalties from Elsevier (editor, *Braunwald’s Heart Disease*); has served as site coinvestigator for Abbott, Biotronik, Boston Scientific, CSI, Endotronix, St Jude Medical (now Abbott), Philips, SpectraWAVE, Svelte, and Vascular Solutions; has served as trustee for American College of Cardiology; and performed unfunded research for FlowCo. Dr Butler has served as a consultant to Abbott, American Regent, Amgen, Applied Therapeutic, AskBio, Astellas, AstraZeneca, Bayer, Boehringer Ingelheim, Boston Scientific, Bristol Myers Squibb, Cardiac Dimension, Cardiocell, Cardior, CSL Bearing, CVRx, Cytokinetics, Daxor, Edwards, Element Science, Faraday, Foundry, G3P, Innolife, Impulse Dynamics, Imbria, Inventiva, Ionis, Lexicon, Lilly, LivaNova, Janssen, Medtronic, Merck, Occlutech, Owkin, Novartis, Novo Nordisk, Pfizer, Pharmacosmos, Pharmain, Prolaio, Regeneron, Renibus, Roche, Salamandra, Sanofi, SC Pharma, Secretome, Sequana, SQ Innovation, Tenex, Tricog, Ultromics, Vifor, and Zoll. Dr Goto reports serving as Associate Editor for *Circulation* and receipt of a steering committee fee from the Duke Clinical Research Institute for EMPACT-MI. Dr Lopes reports research grants or contracts from Amgen, Bristol Myers Squibb, GlaxoSmithKline, Medtronic, Pfizer, and Sanofi-Aventis; funding for educational activities or lectures from Pfizer, Daiichi Sankyo, and Novo Nordisk; and funding for consulting or other services from Bayer, Boehringer Ingelheim, Bristol Myers Squibb, and Novo Nordisk. Dr Amir reports serving as National PI-Steering committee member for the study and participated in paid lectures and advisory board meetings and clinical trials in Dr Amir’s department at Boehringer Ingelheim. Dr Beyes-Genis has lectured or participated in advisory boards for Abbott, AstraZeneca, Bayer, Boehringer-Ingelheim, Medtronic, Novartis, Novo Nordisk, Roche Diagnostics, and Vifor. Dr Bahit reports modest honorarium from MSD, Pfizer, Bristol Myers Squibb, CSL Behring, Janssen, Boehringer Ingelheim, and Anthos Therapeutics. Dr Bauersachs received honoraria for lectures/consulting from Novartis, Vifor, Bayer, Pfizer, Boehringer Ingelheim, AstraZeneca, Cardior, CVRx, BMS, Amgen, Corvia, Norgine, Edwards, and Roche not related to this article; and research support for Dr Bauersachs’ department from Zoll, CVRx, Abiomed, Norgine, and Roche, not related to this article. Dr Schou reports lecture fees from Novartis, Astra Zeneca, Bohringer, and Novo Nordisk. Dr Steg reports research grants from Amarin and Sanofi; clinical trial participation for Amarin, Amgen, AstraZeneca, Idorsia, Janssen, Novartis, Novo-Nordisk, and Sanofi; consulting or speaking for Amarin, Amgen, and Novo-Nordisk; and serving as senior associate editor at *Circulation*. Dr Parikh reports serving as a consultant for Medtronic, Inc and receipt of an institutional research grant from Abbott and Edwards Lifesciences. Dr Januzzi reports participation as a trustee of the American College of Cardiology, board member of Imbria Pharmaceuticals, and director at Jana Care; has received research support from Abbott, Applied Therapeutics, Bayer, BBMS, HeartFlow Inc, Innolife, and Roche Diagnostics, and consulting income from Abbott, AstraZeneca, Bayer, Beckman, Boehringer-Ingelheim, Janssen, Medtronic, Novartis, Prevencio, Quidel/Ortho, Roche Diagnostics, and Vascular Dynamics; and participates in clinical end point committees or data safety monitoring boards for Abbott, AbbVie, Bayer, CVRx, Medtronic, Pfizer, Roche Diagnostics, and Takeda. Dr Goodman reports research grant support (eg, steering committee or data and safety monitoring committee) or speaker or consulting honoraria (eg, advisory boards) from Amgen, Anthos Therapeutics, AstraZeneca, Bayer, Boehringer Ingelheim, Bristol Myers Squibb, CSL Behring, CYTE Ltd, Daiichi-Sankyo/American Regent, Eli Lilly, Esperion, Ferring Pharmaceuticals, HLS Therapeutics, Idorsia, JAMP Pharma, Merck, Novartis, Novo Nordisk A/C, Pendopharm/Pharmascience, Pfizer, Regeneron, Sanofi, Servier, Tolmar Pharmaceuticals, and Valeo Pharma; and salary support or honoraria from the Heart and Stroke Foundation of Ontario/University of Toronto (Polo) Chair, Canadian Heart Failure Society, Canadian Heart Research Centre and MD Primer, Canadian VIGOUR Centre, Cleveland Clinic Coordinating Centre for Clinical Research, Duke Clinical Research Institute, New York University Clinical Coordinating Centre, PERFUSE Research Institute, and TIMI Study Group (Brigham Health). Dr van der Meer reports support from the European Research Council (ERC CoG 101045236, DISSECT-HF); the UMCG, which employs Dr van der Meer, received consultancy fees or grants from Novartis, Pharmacosmos, Vifor Pharma, Astra Zeneca, Pfizer, Pharma Nord, BridgeBio, Novo Nordisk, Daiichi Sankyo, Boehringer Ingelheim, and Ionis. Dr Petrie reports research funding from Boehringer Ingelheim, Roche, SQ Innovations, Astra Zeneca, Novartis, Novo Nordisk, Medtronic, Boston Scientific, and Pharmacosmos; and consultancy or trial committee participation from Akero, Applied Therapeutics, Amgen, AnaCardio, Biosensors, Boehringer Ingelheim, Novartis, Astra Zeneca, Novo Nordisk, AbbVie, Bayer, Horizon Therapeutics, Takeda, Cardiorentis, Pharmacosmos, Siemens, Eli Lilly, Vifor, New Amsterdam, Moderna, Teikoku, LIB Therapeutics, and 3R Lifesciences. Dr Parkhomenko reports research grants and personal fees from Bayer, Amgen, Astra Zeneca, Boehringer Ingelheim, BMS/Pfizer, and Daiichi-Sankyo. Dr Vinereanu reports research grants and consultancy fees from Boehringer Ingelheim and research grants from Bayer Healthcare, Novartis, and Servier Pharmaceuticals LLC. Dr Zieroth reports research grant support, served on advisory boards for, or had speaker engagements with AstraZeneca, Bayer, BMS, Boehringer Ingelheim, Cytokinetics, Eli Lilly, GSK, Janssen, Medtronic, Merck, Novartis, Novo Nordisk, Otsuka, Pfizer, Roche, Salubrisbio, Servier, and Vifor Pharma; and serves on a clinical trial committee or as a national lead for studies sponsored by AstraZeneca, Bayer, Boehringer Ingelheim, Merck, Novartis and Pfizer; nonindustry participation includes Canadian Medical and Surgical KT Group, CCS, CHFS, Charite, EOCI, Liv, Medscape, Ology, PACE-CME, Radcliffe, Reach MD, and Translational Medicine Academy. Dr Jones reports research grants from Bayer, Boehringer Ingelheim, Merck, Novartis, PCORI, and the National Institutes of Health. Drs Seide, Mattheus, Zwiener, Sumin, Gasior, Jamal, and Brueckmann are employees of Boehringer Ingelheim.

### Supplemental Material

Appendix

Major protocol-specified efficacy end points

Outcome events definitions and verification in the trial

Collection of adverse events

Analysis of heart failure adverse events

Tables S1–S4

Figures S1–S6

## Supplementary Material


