# Supplementary material for: Effect of Empagliflozin on Heart Failure Outcomes After Acute Myocardial Infarction: Insights From the EMPACT-MI Trial
Source: Circulation. 2024 Apr 6;149(21):1627–38. doi: 10.1161/CIRCULATIONAHA.124.069217 (PMC11115458; doi:10.1161/CIRCULATIONAHA.124.069217)
Supplement: Supplementary file 1 [file cir-149-01627-s001.pdf]

SUPPLEMENTAL MATERIAL

Contents

MAJOR PROTOCOL-SPECIFIED EFFICACY ENDPOINTS 2

OUTCOME EVENTS DEFINITIONS AND VERIFICATION IN THE TRIAL 2

COLLECTION OF ADVERSE EVENTS 6

ANALYSIS OF HEART FAILURE ADVERSE EVENTS 6

SUPPLEMENTARY TABLE 1. PRE-SPECIFICATION OF OUTCOMES AND STATISTICAL ANALYSES IN THE EMPACT-MI TRIAL 8

SUPPLEMENTARY TABLE 2. BASELINE CHARACTERISTICS BASED ON ALL PATIENTS RANDOMIZED 10

SUPPLEMENTARY TABLE 3. NUMBER AND FREQUENCY OF ADVERSE EVENTS OF HEART FAILURE (BASED ON NARROW SMQ CARDIAC FAILURE) BY PREFERRED TERM BASED ON ALL PATIENTS RANDOMIZED 12

SUPPLEMENTARY TABLE 4. NUMBER OF ADVERSE EVENTS OF HEART FAILURE (NARROW SMQ CARDIAC FAILURE) AND BREAKDOWN OF ALL EVENTS INTO SAE CRITERIA BASED ON ALL PATIENTS RANDOMIZED 13

SUPPLEMENTARY FIGURE 1. HEART FAILURE ENDPOINTS ANALYSES METHODOLOGY AND DETAILS OF DATA COLLECTION 15

SUPPLEMENTARY FIGURE 2: SENSITIVITY ANALYSES FOR TIME TO FIRST HOSPITALIZATION FOR HEART FAILURE AND TOTAL NUMBER OF HOSPITALIZATIONS FOR HEART FAILURE 16

SUPPLEMENTARY FIGURE 3. MAJOR HEART FAILURE AND MORTALITY OUTCOMES (I) 16

SUPPLEMENTARY FIGURE 4. MAJOR HEART FAILURE AND MORTALITY OUTCOMES (II) 17

SUPPLEMENTARY FIGURE 5: ADVERSE EVENTS OF HEART FAILURE (I) 18

SUPPLEMENTARY FIGURE 6. ADVERSE EVENTS OF HEART FAILURE (II) 19

MAJOR PROTOCOL-SPECIFIED EFFICACY ENDPOINTS

Primary endpoint

The primary endpoint is the composite of time to first heart failure hospitalization (HHF) or all-cause mortality, calculated from date of randomisation.

Secondary endpoint(s)

Key secondary endpoints, which are part of the confirmatory testing strategy, are:

- Total number of HHF or all-cause mortality
- Total number of non-elective cardiovascular (CV) hospitalizations or all-cause mortality
- Total number of non-elective all-cause hospitalizations or all-cause mortality
- Total number of hospitalizations for MI or all-cause mortality

Other secondary endpoints, not part of the confirmatory testing strategy, are:

- Time to CV mortality

OUTCOME EVENTS DEFINITIONS AND VERIFICATION IN THE TRIAL

The trial didn't use conventional central adjudication committee.
The algorithm of hospitalization and death events verification in the trial included the following components:

- Blinded investigators were responsible for endpoints collection, reviewing appropriate clinical details and categorization according to the definitions provided below. All investigators received training in event review as a prerequisite for trial qualification. This training included instruction on the accurate assessment and documentation of clinical endpoints, adverse and serious adverse events, and adverse events of special interest.
- Information about hospitalizations and adverse events was entered by the investigators into structured eCRF pages which allowed to verify whether the events were fulfilling prespecified endpoints definition (see below) during data entry process.
- Verification of the data entered in eCRF against source documents was performed within source data verification and source data review process according to trial monitoring plan
- Automatic data check for data completeness
- Medical and data management review of endpoints information entered in eCRF
- For the primary analysis, only endpoints which fulfilled the pre-specified criteria as per event review charter have been included as endpoints
- To investigate the influence of investigator-based collection of HHF events, sensitivity analyses have been performed on the composite of time to first investigator-reported broad HHF and total number of investigator-reported broad HHF, using the definition of HHF as per event review charter and additionally including hospitalizations with criteria outlined in the event review charter but:
    a. irrespective of electivity of the corresponding hospitalization
    b. irrespective of the presence of signs, symptoms or laboratory findings of HF
    c. irrespective of treatment for HF
    d. irrespective of all of electivity, presence of signs, symptoms and laboratory findings and treatment for HF (combination of a, b and c)

Outcome events definitions used in the trial

<u>HOSPITALIZATIONS</u>

Hospitalizations are key endpoints in EMPACT-MI. All hospitalizations were to be collected and reported during the trial. To qualify as a hospitalization, the hospital stay had to include at least one date change (i.e. at least one overnight stay) to be analysed as a hospitalization. For all hospitalizations, admission and discharge date and information on whether elective or non-elective was collected. For certain endpoints specified in the trial protocol, only the non-elective hospitalizations were analysed (as pre-specified in the event review charter).

These are HHF as part of primary and key secondary endpoints, CV, and all-cause hospitalizations as part of key secondary endpoints.

Hospitalization for Heart Failure (HHF)
HHF is one of the components of the primary endpoint. Detailed information related to these events was collected on the Hospitalization page. For analysis as a HHF in EMPACT-MI (for primary and key secondary endpoints), hospitalizations had to be non-elective with a primary cause of heart failure, defined as at least one heart failure symptom or sign (lab and imaging findings are considered as signs) requiring treatment (e.g. diuretics, inotropes, mechanical circulatory support).

To ensure that all HHF events were properly captured it was critical that sites had to obtain supporting source documentation, likely to include the above information. At minimum, this typically had to include the admission note and discharge summary, but additional supporting information was supposed be found in progress notes, procedure reports (e.g. right heart catheterization report), laboratory data (e.g. BNP or NT-proBNP pro-NT BNP), and other medical notes.

HHF components

- Non-elective/unplanned: The designation of non-elective/unplanned is typically referenced in the source data. Examples of non-elective/unplanned include:
  - Hospitalization from emergency department
  - Hospitalizations where patient was admitted directly to the ICU/medical ward from home due to worsening HF
  - Hospitalizations where patient was admitted directly to the ICU/medical ward from outpatient clinic due to worsening HF
  - Hospitalizations that were initially planned but where worsening HF complicated and prolonged hospital stay (e.g. admission for planned cardiac procedure but worsening HF evident requiring prolonged hospitalization)

- Symptoms, including all symptoms that were related to HF, which lead to hospitalization. If a symptom of HF was not represented in the preselection in eCRF, option ‘Other’ had to be selected.

- Signs, includes either physical or lab/imaging findings consistent with HF:

  - Physical signs related to HF, typically evident at presentation or early during the hospitalization. If a sign of HF was not represented in the eCRF preselection, option ‘Other’ had to be selected.
  - Lab/imaging findings consistent with worsening HF, incl. elevated natriuretic peptides, radiological evidence of congestion etc. Lab/imaging findings might not always be available immediately upon admission (for instance chest x-ray performed day after admission). If a lab/imaging finding corresponding to HF was not represented in the eCRF preselection, option ‘Other’ had to be selected.

- Treatment: includes any of the defined treatments for worsening HF (i.e. initiated or intensified oral or iv diuretics, other iv HF therapies, mechanical fluid removal or circulatory support) administered during the hospitalization.

Hospitalization for myocardial infarction

Hospitalization for MI is a component of a key secondary endpoint. These are hospitalizations with a primary cause of acute myocardial infarction, defined according to prevailing guidelines. Given the acute nature of this hospitalization, it had never to be recorded as ‘elective’. In the eCRF, it was further classified as either non-ST elevation MI or ST-elevation MI based on best available source documentation (e.g. ECG, medical notes/discharge summary).

Hospitalization for any other CV reason

A hospitalization with a primary cause as 'any other CV' encompassed hospitalizations with a primary CV reason other than HF or MI. This included, but was not limited to, ischemic heart disease (other than MI), arrhythmias, valvular heart disease, aortic dissection or rupture, peripheral artery disease events and pulmonary embolism.

Hospitalization for non-CV reason
Any hospitalization that did not meet the criteria for HF, MI or any other CV hospitalization and had a non-CV reason as the primary cause for hospitalization.

Death
All-cause mortality is a component of the primary composite endpoint. Similar to traditional clinical studies, investigators were asked to judge and report the most likely cause of death. For all deaths, sites collected and investigators reviewed all available information including hospital records (discharge or death summaries), death certificates, autopsy reports and reports from potential witnesses and relatives. Investigators then used this information to confirm and report the primary cause of death using the convention "If not for (blank), the participant would still be alive", in which case (blank) would be the cause of death. For in-hospital deaths where the primary cause couldn't be easily determined by any of the below descriptions, the cause of hospital admission was recommended also to be considered the primary cause of death.

The following death subcategories were reported in the eCRF:

Cardiovascular (CV) death

- **Acute myocardial infarction**
  - Typically, death due to acute MI was considered any death within 30 days after a confirmed MI if related to the immediate consequences of the MI (e.g. deaths due to heart failure, sudden cardiac death following acute MI).
  - Acute MI had to be verified with source data (hospitalization summary, troponin values, and/or autopsy) and there should not be other explanations for death (e.g. trauma, non-CV causes).
  - In the event that patients present with symptoms of acute MI, have ECG/angiography/autopsy evidence of acute MI, but do not have troponin assays, these deaths should be classified as due to acute MI.

- **Sudden Cardiac Death**
  - Sudden cardiac death was defined as any death that occurred unexpectedly.
  - Witnessed deaths occurring without any new or worsening symptoms; or within 60 minutes of new or worsening symptoms (but not consistent with acute MI as above).
  - Unwitnessed deaths where patient was seen alive and clinically stable <24 hours and there is no evidence supporting other likely cause.
  - Any death after unsuccessful resuscitation from cardiac arrest and without other specific CV- or non-CV cause specified in this document (i.e. known MI, HF, other CV or non-CV death).

- **Heart Failure**
  - Death associated with clinically worsening symptoms and/or signs of heart failure (regardless of HF etiology) and also included deaths that resulted from complications of treatments for heart failure (e.g. LVAD, heart transplant).
  - Deaths that occurred during a heart failure hospitalization were generally be attributed to heart failure, even if there were another immediate CV cause of death (e.g., ventricular fibrillation).
  - Deaths that occurred in hospice or other similar palliative care setting for heart failure was to be attributed to heart failure.

- **Other CV Causes**
  - Death due to Other CV Causes include any deaths with cardiovascular cause other than sudden cardiac death, MI or HF; including stroke, CV procedure, CV hemorrhage etc. There were no subclassification of other CV Causes of death.

Non-CV death: there was no subclassification of non-CV death.

Unknown cause of death
Uncertainty could remain after review of available evidence, and while rare, Unknown Cause of Death had only be selected in cases where minimal or no information related to the death was available to determine a likely cause. In order to minimize the selection of Unknown Cause of Death, sites had to make every effort to gather source data (when it exists) and narratives from family members/friends/neighbours.

Other specific events/procedures
In addition to death and hospitalisation, a few additional events/procedures needed to be collected and recorded on the Concomitant Non-drug Therapy page. These were to be recorded irrespective of whether they were part of a hospitalization or not, and included:

- myocardial revascularization procedures (percutaneous coronary intervention or coronary artery bypass grafting)
- cardiac device procedures (implantable cardioversion defibrillator or cardiac resynchronization therapy)
- renal replacement therapy/dialysis and renal transplantation

COLLECTION OF ADVERSE EVENTS

Adverse events collection in the trial

The clinical trial protocol specified that all adverse events of special interest (serious and non-serious), serious adverse events and adverse leading to discontinuation of trial medication for at least 7 consecutive days (serious and non-serious) from randomisation onwards until the individual patient's end of trial participation had to be collected and documented on the appropriate eCRF(s) by the investigator.

For the trial specific definition of adverse events, please refer to the Supplementary appendix of Butler J. et al[8].

As per clinical trial protocol serious adverse event was defined as any adverse event, which fulfils at least one of the following criteria:

- results in death,
- is life-threatening, which refers to an event in which the patient was at risk of death at the time of the event; it does not refer to an event that hypothetically might have caused death if more severe,
- requires inpatient hospitalisation or prolongation of existing hospitalisation
- results in persistent or significant disability or incapacity,
- is a congenital anomaly / birth defect,
- is deemed serious for any other reason if it is an important medical event when based on appropriate medical judgement which may jeopardise the patient and may require medical or surgical intervention to prevent one of the other outcomes listed in the above definitions. Examples of such events (as provided in the clinical trial protocol) are intensive treatment in an emergency room or at home for allergic bronchospasm, blood dyscrasias or convulsions that do not result in hospitalisation or development of dependency or abuse.

Adverse events considered "Always Serious"

The clinical trial protocol specified the following details for reporting of always serious adverse events.
In accordance with the European Medicines Agency initiative on Important Medical Events, Boehringer Ingelheim has set up a list of adverse events, which by their nature, can always be considered to be "serious" even though they may not have met the criteria of a serious adverse event as defined above.
The list of "Always Serious Adverse events" was made available to the site via the electronic data capture system.
These events should always have been reported as serious adverse events.

This list of "Always Serious Adverse events" includes the following preferred terms (based on MedDRA version 26.1), that are part of the narrow SMQ cardiac failure and thereby all adverse events corresponding to these preferred terms had to be reported in this clinical trial, and had additionally to be classified as serious:

Cardiac failure, Cardiac failure congestive, Cardiac failure acute, Acute left ventricular failure, Acute right ventricular failure, Acute pulmonary oedema, Cardiogenic shock, Cardiohepatic syndrome, Cardiopulmonary failure, Cardiorenal syndrome, Cor pulmonale, Cor pulmonale acute, Cor pulmonale chronic, Left ventricular failure, Low cardiac output syndrome, Obstructive shock, Pulmonary oedema, Pulmonary oedema neonatal, Right ventricular failure, Ventricular failure.

ANALYSIS OF HEART FAILURE ADVERSE EVENTS

For the analysis of adverse events of heart failure we used the narrow standardized MedDRA query (SMQ) "cardiac failure" (MedDRA 26.1), which contains the following preferred terms: Cardiac failure, Cardiac failure congestive, Cardiac failure acute, Acute left ventricular failure, Acute right ventricular failure, Acute pulmonary oedema, Cardiogenic shock, Cardiohepatic syndrome, Cardiopulmonary failure, Cardiorenal syndrome, Cor pulmonale, Cor pulmonale acute, Cor pulmonale chronic, Left ventricular failure, Low cardiac output syndrome,

Obstructive shock, Pulmonary oedema, Pulmonary oedema neonatal, Right ventricular failure, Ventricular failure, Cardiac asthma, Cardiac failure chronic, Cardiac failure high output, Chronic left ventricular failure, Chronic right ventricular failure, Congestive hepatopathy, Ejection fraction decreased, Hepatojugular reflux, Neonatal cardiac failure, Radiation associated cardiac failure, Right ventricular ejection fraction decreased.

The list of PTs of MedDRA narrow SMQ ‘of cardiac failure’ that were not on the list to be always reported in the trial as serious adverse events and hence not required to be reported at all (unless leading to treatment discontinuation for >= 7 consecutive days or other seriousness criteria fulfilled): Cardiac asthma, Cardiac failure chronic, Cardiac failure high output, Chronic left ventricular failure, Chronic right ventricular failure, Congestive hepatopathy, Ejection fraction decreased, Hepatojugular reflux, Neonatal cardiac failure, Radiation associated cardiac failure, Right ventricular ejection fraction decreased.

Supplementary Table 1 outlines the preferred terms of reported heart failure adverse events based on narrow SMQ cardiac failure in the trial.
All actually reported adverse events within narrow SMQ cardiac failure were reported as serious adverse events. See details of the seriousness criteria in Supplementary Table 2.

The time to first onset of adverse events of heart failure was defined by the adverse event onset date. For the total number of adverse events of heart failure based on the narrow SMQ cardiac failure we counted the total number of adverse events within this SMQ with distinct onset dates. For time to first onset of event of heart failure or all-cause mortality the first as of onset date of the adverse event or date of death was used. For total number of events of heart failure or all-cause mortality we counted the total number of events with distinct onset dates and all deaths.

For the analysis of heart failure events requiring/prolonging hospitalization or with fatal outcome we additionally required at least one of the respective serious adverse event criteria of ‘requires hospitalization or prolongation of existing hospitalization’ or ‘results in death’ to be fulfilled for an event to qualify for these endpoints.

For the analysis of outpatient non-fatal events of heart failure we additionally required that none of the two serious adverse event criteria of ‘requires hospitalization or prolongation of existing hospitalization’ and ‘results in death’ were fulfilled for an event to qualify for these endpoints.

SUPPLEMENTARY TABLE 1. PRE-SPECIFICATION OF OUTCOMES AND STATISTICAL ANALYSES IN THE EMPACT-MI TRIAL

	Pre-specified (prior to database lock)	
	First event analysis	Total (first plus recurrent) event analysis
Outcome:		
HHF	Yes	Yes
HHF or CV death	Yes	Yes
HHF or death due to Heart Failure	No	No
HHF additionally including hospitalisation irrespective of electivity of the corresponding hospitalisation	No (only pre-specified for HHF component as part of primary endpoint)	No (only pre-specified for HHF component as part of first key secondary endpoint)
HHF additionally including hospitalisation irrespective of the presence of signs, symptoms or laboratory findings	No (only pre-specified for HHF component as part of primary endpoint)	No (only pre-specified for HHF component as part of first key secondary endpoint)
HHF additionally including hospitalisation irrespective of treatment for Heart Failure	No (only pre-specified for HHF component as part of primary endpoint)	No (only pre-specified for HHF component as part of first key secondary endpoint)
HHF additionally including hospitalisation irrespective of electivity, presence of signs, symptoms and laboratory findings and treatment for Heart Failure	No (only pre-specified for HHF component as part of primary endpoint)	Yes
Adverse event of heart failure (narrow SMQ ‘cardiac failure’)	No	No
Adverse event of heart failure (narrow SMQ ‘cardiac failure’)or CV death	No	No
Adverse event of heart failure (narrow SMQ ‘cardiac failure’)or all-cause mortality	No	No
Adverse event of heart failure (narrow SMQ ‘cardiac failure’)requiring or prolonging hospitalisation or with fatal outcome	No	No
Outpatient non-fatal adverse event of heart failure*	No	No
New use of diuretics[#] until 6 months from discharge (in patients not on diuretics at discharge)	No	–
New use of ARNI until 6 months from discharge (in patients not on ARNI at discharge)	No	–
New use of ACE inhibitors, ARB or ARNI until 6 months from discharge (in patients not on ACE inhibitors, ARB or ARNI at discharge)	No	–
New use of MRA until 6 months from discharge (in patients not on MRA at discharge)	No	–
Time to first implantation of either implantable cardiac defibrillator (ICD) and/or cardiac resynchronization therapy (CRT) device	Yes	–
Statistical models:		
Cox regression model	Yes	–
Negative binomial regression model	–	Yes
WLW model	–	Yes
Joint Frailty model (with all-cause mortality as competing risk)	–	Yes

	Pre-specified (prior to database lock)	
	First event analysis	Total (first plus recurrent) event analysis
Kaplan Meier or Cumulative incidence function curves	Yes	–
Mean cumulative function curves	–	Yes
Other analyses:		
Sensitivity analyses of HHF with model including only treatment and stratification factors (region and diabetes status) as covariates	No (only pre-specified for primary and key secondary endpoints)	No (only pre-specified for primary and key secondary endpoints)
Subgroup analyses for HHF (according to pre-specified subgroups)	Yes	Yes (partly)

* Defined as adverse events of heart failure (narrow SMQ 'cardiac failure')that neither result in death nor leading/prolonging hospitalisation
#Other than MRA
HHF=hospitalisation for heart failure. CV=Cardiovascular. ACE= Angiotensin-converting-enzyme, ARB=Angiotensin receptor blockers, ARNI= Angiotensin receptor-neprilysin inhibitors, MRA=Mineralocorticoid receptor antagonists, WLW=Wei-Lin-Weissfeld.

SUPPLEMENTARY TABLE 2. BASELINE CHARACTERISTICS BASED ON ALL PATIENTS RANDOMIZED

Characteristic	Empagliflozin (N=3260)	Placebo (N=3262)	Total (N=6522)
Age (years)	63.6 (11.0)	63.7 (10.8)	63.6 (10.9)
Women - no. (%)	812 (24.9%)	813 (24.9%)	1625 (24.9%)
Race - no. (%)[§]			
White	2730 (83.7%)	2721 (83.4%)	5451 (83.6%)
Black/African American	44 (1.3%)	48 (1.5%)	92 (1.4%)
Asian	421 (12.9%)	413 (12.7%)	834 (12.8%)
Other or missing	65 (2.0%)	80 (2.4%)	145 (2.2%)
Region – no. (%)			
North America	431 (13.2%)	433 (13.3%)	864 (13.2%)
Latin America	290 (8.9%)	288 (8.8%)	578 (8.9%)
Europe	2153 (66.0%)	2154 (66.0%)	4307 (66.0%)
Asia	386 (11.8%)	387 (11.9%)	773 (11.9%)
Index myocardial infarction type – no. (%)[§]			
ST elevation	2444 (75.0%)	2401 (73.6%)	4845 (74.3%)
Non-ST elevation	814 (25.0%)	861 (26.4%)	1675 (25.7%)
Revascularization for index myocardial infarction – no. (%)	2911 (89.3%)	2911 (89.2%)	5822 (89.3%)
Thrombolytic therapy for index myocardial infarction – no. (%)	345 (10.6%)	355 (10.9%)	700 (10.7%)
Time from index MI diagnosis to revascularization – median in days (IQR)	0 (0, 0)	0 (0, 0)	0 (0, 0)
Lowest left ventricular ejection fraction – no. (%)[§]			
<25%	126 (3.9%)	126 (3.9%)	252 (3.9%)
≥25%- <35%	721 (22.1%)	699 (21.4%)	1420 (21.8%)
≥35%- <45%	1724 (52.9%)	1716 (52.6%)	3440 (52.7%)
≥45%-<55%	438 (13.4%)	468 (14.3%)	906 (13.9%)
≥55%	227 (7.0%)	225 (6.9%)	452 (6.9%)
Signs/symptoms of congestion requiring treatment – no. (%)	1852 (56.8%)	1863 (57.1%)	3715 (57.0%)
With lowest LVEF <45%[§]	1172 (36.0%)	1151 (35.3%)	2323 (35.6%)
With lowest LVEF >=45%[§]	657 (20.2%)	684 (21.0%)	1341 (20.6%)
Number of additional enrichment factors[$] – no. (%)			
≥2 factors	2325 (71.3%)	2273 (69.7%)	4598 (70.5%)
≥3 factors	1363 (41.8%)	1405 (43.1%)	2768 (42.4%)
≥4 factors	700 (21.5%)	722 (22.1%)	1422 (21.8%)
Cardiovascular history – no. (%)			
Prior myocardial infarction	388 (11.9%)	459 (14.1%)	847 (13.0%)
Atrial fibrillation	358 (11.0%)	361 (11.1%)	719 (11.0%)
Type-2 diabetes mellitus	1035 (31.7%)	1046 (32.1%)	2081 (31.9%)
Hypertension	2262 (69.4%)	2276 (69.8%)	4538 (69.6%)
Peripheral arterial disease	172 (5.3%)	180 (5.5%)	352 (5.4%)
Baseline Estimated glomerular filtration rate (eGFR)			

Median, interquartile range (ml/min/1.73 m^2)	77.5 (62.2-91.0)	78.0 (61.7-91.4)	77.8 (61.9-91.2)
No. (%) with eGFR <60 ml/min/1.73 m^2	720 (22.1%)	738 (22.6%)	1458 (22.4%)
Body mass index (kg/m^2)[§]	28.1 (5.0)	28.1 (5.0)	28.1 (5.0)
Heart rate (beats/min)[§]	73.2 (11.1)	73.7 (11.5)	73.5 (11.3)
Systolic blood pressure (mmHg)[§]	120.3 (14.6)	120.5 (15.2)	120.4 (14.9)
Key cardiovascular medications at discharge from index hospitalization - no. (%)			
ACE inhibitor, angiotensin receptor blocker, or angiotensin receptor neprilysin inhibitor	2683 (82.3%)	2698 (82.7%)	5381 (82.5%)
Angiotensin receptor neprilysin inhibitor	218 (6.7%)	236 (7.2%)	454 (7.0%)
Diuretics	2125 (65.2%)	2125 (65.1%)	4250 (65.2%)
Diuretics other than MRAs	1472 (45.2%)	1389 (42.6%)	2861 (43.9%)
Loop Diuretics	1301 (39.9%)	1198 (36.7%)	2499 (38.3%)
Mineralocorticoid receptor antagonist	1542 (47.3%)	1575 (48.3%)	3117 (47.8%)
Beta-blocker	2820 (86.5%)	2835 (86.9%)	5655 (86.7%)
Dual antiplatelet therapy*	2942 (90.2%)	2951 (90.5%)	5893 (90.4%)
Dual antiplatelet or antiplatelet plus anticoagulation therapy**	3067 (94.1%)	3056 (93.7%)	6123 (93.9%)
Statin	3110 (95.4%)	3107 (95.2%)	6217 (95.3%)
Key cardiovascular medications at baseline - no. (%)			
ACE inhibitor, angiotensin receptor blocker, or Angiotensin receptor neprilysin inhibitor	2395 (73.5%)	2331 (71.5%)	4726 (72.5%)
Angiotensin receptor neprilysin inhibitor	158 (4.8%)	171 (5.2%)	329 (5.0%)
Diuretics	1823 (55.9%)	1853 (56.8%)	3676 (56.4%)
Diuretics other than MRAs	1272 (39.0%)	1241 (38.0%)	2513 (38.5%)
Loop Diuretics	1134 (34.8%)	1078 (33.0%)	2212 (33.9%)
Mineralocorticoid receptor antagonist	1268 (38.9%)	1305 (40.0%)	2573 (39.5%)
Beta-blocker	2515 (77.1%)	2534 (77.7%)	5049 (77.4%)
Dual antiplatelet therapy*	2768 (84.9%)	2752 (84.4%)	5520 (84.6%)
Dual antiplatelet or antiplatelet plus anticoagulation therapy**	2859 (87.7%)	2840 (87.1%)	5699 (87.4%)
Statin	2907 (89.2%)	2891 (88.6%)	5798 (88.9%)

Where not stated, data present mean (standard deviation).

[#] At discharge of index hospitalization

*Includes patients receiving dual antiplatelet therapy (P2Y12 inhibitor and acetylsalicylic acid)

** Includes patients receiving dual antiplatelet therapy or at least one antiplatelet agent and oral anticoagulant (Vitamin K antagonist or direct oral anticoagulant)

$ Enrichment criteria included age ≥65 years, LVEF <35%, eGFR <60 ml/min/1.73m2, prior myocardial infarction, atrial fibrillation, T2D, elevated natriuretic peptide levels (≥1400 pg/ml in sinus rhythm and ≥2800 pg/ml if in atrial fibrillation), uric acid ≥7.5mg/dl, pulmonary artery systolic pressure ≥40 mmHg, no revascularization for the index myocardial infarction, three-vessel coronary artery disease at the time of index myocardial infarction, or peripheral artery disease. Except for eGFR, laboratory values and pulmonary artery pressure have been optional to be reported beyond meeting inclusion criterion of providing at least 1 enrichment criterion.

[§] Number of patients with missing data for race: N=56 in empagliflozin, N=73 in placebo; for index myocardial infarction type: N=2 in empagliflozin, N=0 in placebo; lowest left ventricular ejection fraction: N=24 in

empagliflozin, N=28 in placebo; baseline BMI: N=9 in empagliflozin, N=18 in placebo; baseline heart rate: N=1 in empagliflozin, N=1 in placebo; baseline SBP: N=1 in empagliflozin, N=0 in placebo.

SUPPLEMENTARY TABLE 3. NUMBER AND FREQUENCY OF ADVERSE EVENTS OF HEART FAILURE (BASED ON NARROW SMQ CARDIAC FAILURE) BY PREFERRED TERM BASED ON ALL PATIENTS RANDOMIZED

Preferred term	Placebo<br>N (%)	Empagliflozin<br>N (%)
Number of patients	3262	3260
Number of adverse events	354 (100.0)	227 (100.0)
Cardiac failure	353 (99.7)	226 (99.6)
Cardiogenic shock	1 (0.3)	1 (0.4)
Acute pulmonary edema	1 (0.3)	0 (0.0)

SUPPLEMENTARY TABLE 4. NUMBER OF ADVERSE EVENTS OF HEART FAILURE (NARROW SMQ CARDIAC FAILURE) AND BREAKDOWN OF ALL EVENTS INTO SAE CRITERIA BASED ON ALL PATIENTS RANDOMIZED

	Placebo N (%)	Empagliflozin N (%)
Number of patients	3262	3260
Number of AEs	354	227
Number of serious AEs	354 (100.0)	227 (100.0)
Seriousness criteria*		
Results in death	30 (8.5)	20 (8.8)
Is life-threatening	33 (9.3)	31 (13.7)
Persist or significant disability/incapacity	3 (0.8)	0 (0.0)
Requires or prolongs hospitalization	267 (75.4)	180 (79.3)
Congenital anomaly or birth defect	0 (0.0)	0 (0.0)
Other medically important serious event	103 (29.1)	49 (21.6)
Results in death or requires or prolongs hospitalization*	273 (77.1)	187 (82.4)
Neither results in death nor requires or prolongs hospitalization**	81 (22.9)	40 (17.6)
1 seriousness criteria		
Other medically important serious event	75 (21.2)	37 (16.3)
Requires or prolongs hospitalization	205 (57.9)	141 (62.1)
Persist or significant disability/incapacity	1 (0.3)	0 (0.0)
Is life-threatening	5 (1.4)	3 (1.3)
Results in death	4 (1.1)	3 (1.3)
2 seriousness criteria		
Requires or prolongs hospitalization and other medically important serious event	22 (6.2)	9 (4.0)
Is life-threatening and requires or prolongs hospitalization	11 (3.1)	15 (6.6)
Results in death and requires or prolongs hospitalization	12 (3.4)	5 (2.2)
Results in death and is life-threatening	2 (0.6)	4 (1.8)
3 seriousness criteria		
Is life-threatening and requires or prolongs hospitalization and other medically important serious event	3 (0.8)	2 (0.9)
Is life-threatening and persist or significant disability/incapacity and requires or prolongs hospitalization	1 (0.3)	0 (0.0)
Results in death and requires or prolongs hospitalization and other medically important serious event	2 (0.6)	1 (0.4)
Results in death and is life-threatening and requires or prolongs hospitalization	10 (2.8)	7 (3.1)
4 seriousness criteria		

Is life-threatening and persist or significant disability/incapacity and requires or prolongs hospitalization and other medically important serious event	1 (0.3)	0 (0.0)

*Multiple selections are possible.

**May include one or multiple selections, but excludes all events that have as one/at least as one option ‘results in death’ or ‘requires or prolongs hospitalization’.

AE, adverse event; SAE, serious adverse event; SMQ, standardized MedDRA queries.
.

SUPPLEMENTARY FIGURE 1. HEART FAILURE ENDPOINTS ANALYSES METHODOLOGY AND DETAILS OF DATA COLLECTION

Endpoint definition	Mechanics of reporting	Data collected
**HOSPITALIZATION FOR HEART FAILURE**		
Time to first and total number of hospitalizations for heart failure (HHF) is a pre-specified trial endpoint for which HHF is defined as a hospitalization with primary cause of heart failure which is:<br>• non-elective<br>• with at least one heart failure symptom or sign (lab and imaging findings are considered as signs)<br>• requiring treatment (e.g. diuretics, inotropes, mechanical circulatory support)<br>• has at least one day change between admission and discharge	The blinded and trained investigators evaluated and categorized hospitalization events according to events review charter and entered the data into structured eCRF hospitalization form<br>**Only the events that met the criteria of the Hospitalization for Heart Failure prespecified in the events review charter where included in the analysis of the endpoints of time to first and total number of HHF**	The information collected for each event of hospitalization with primary cause of heart failure: date of admission; date of discharge; hospitalization non-elective or elective/planned; primary cause for hospitalization; corresponding adverse event; new or worsening symptoms, signs or laboratory findings of HF (incl. details); was therapy for heart failure initiated or intensified (incl. details)
**ADVERSE EVENTS OF HEART FAILURE**		
Time to first and total number of adverse events of HF (narrow SMQ cardiac failure) is a post-hoc established endpoint for which adverse event of heart failure is defined as an adverse event within MedDRA narrow SMQ "Cardiac Failure", collected in the trial<br>The adverse events of HF collected in the trial were categorized to:<br>• adverse events of HF requiring hospitalization/prolongation of hospitalization or fatal events (using SAE seriousness criteria and SAE outcome)<br>• outpatient events (those not resulting in hospitalization, prolongation of hospitalization and non-fatal, using SAE seriousness criteria and SAE outcome)	Events reporting via adverse events form<br>The adverse events to be reported in the trial included: adverse events of special interest, adverse events leading to treatment discontinuation for ≥7 consecutive days, SAEs<br>An SAE is defined as any adverse event that is fatal, life-threatening, resulting in hospitalization or prolongation of hospitalization, resulting in persistent or significant disability or incapacity, is a congenital anomaly/birth defect, is deemed serious for any other reason, including any adverse events that are "always serious"<br>The list of adverse events which had ALWAYS to be reported as SAE was prespecified as the "List of Always Serious Adverse Events". The list of Always Serious Adverse events included PTs of MedDRA narrow SMQ "Cardiac Failure"*<br>**Therefore, the adverse events collected in the trial include all medical occurrences consistent with the subset of PTs of SMQ "Cardiac Failure" that are included in the List of Always Serious Adverse Events**	**The information collected for each adverse event:** start date, end date, seriousness, criteria of seriousness, action taken with study drug, outcome, therapy, relationship to study treatment; where applicable: details on SAE case form (as per clinical trial protocol)

*The list of the PTs of MedDRA narrow SMQ 'cardiac failure' to be always reported in the trial as serious adverse events: Cardiac failure, Cardiac failure congestive, Cardiac failure acute, Acute left ventricular failure, Acute right ventricular failure, Acute pulmonary oedema, Cardiogenic shock, Cardiohepatic syndrome, Cardiopulmonary failure, Cardiorenal syndrome, Cor pulmonale, Cor pulmonale acute, Cor pulmonale chronic, Left ventricular failure, Low cardiac output syndrome, Obstructive shock, Pulmonary oedema, Pulmonary oedema neonatal, Right ventricular failure, Ventricular failure.
The list of PTs of MedDRA narrow SMQ 'of cardiac failure' that were not on the list to be always reported in the trial as serious adverse events and hence not required to be reported at all (unless leading to treatment discontinuation for >= 7 consecutive days or other seriousness criteria fulfilled): Cardiac asthma, Cardiac failure chronic, Cardiac failure high output, Chronic left ventricular failure, Chronic right ventricular failure, Congestive hepatopathy, Ejection fraction decreased, Hepatojugular reflux, Neonatal cardiac failure, Radiation associated cardiac failure, Right ventricular ejection fraction decreased.
SAE=Serious adverse event, PT=preferred term

SUPPLEMENTARY FIGURE 2: SENSITIVITY ANALYSES FOR TIME TO FIRST HOSPITALIZATION FOR HEART FAILURE AND TOTAL NUMBER OF HOSPITALIZATIONS FOR HEART FAILURE

Variable	Empagliflozin (n=3260)		Placebo (n=3262)		Hazard ratio or rate ratio‡
	N events/ N analysed*	Events/ 100 py†	N events/ N analysed*	Events/ 100 py†	
**Time to first HHF**					
Overall	118/3260	2.6	153/3262	3.4	0.77 (0.60, 0.98)
Sensitivity analyses					
Including only stratification factors as covariates	118/3260	2.6	153/3262	3.4	0.77 (0.60, 0.98)
**Total HHF**					
Overall	148/3260	2.4	207/3262	3.6	0.67 (0.51, 0.89)
Joint frailty model	148/3260	3.2	207/3262	4.4	0.71 (0.55, 0.91)
Sensitivity analyses					
Including only stratification factors as covariates	148/3260	3.3	207/3262	4.8	0.69 (0.52, 0.92)

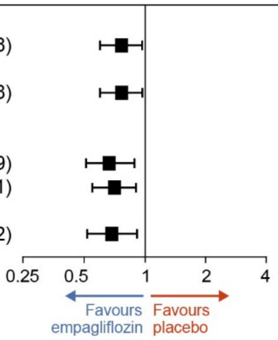

‡Hazard Ratio (95% confidence interval), p-value based on Cox proportional hazards model for time to first event endpoints, Event Rate Ratio (95% CI), based on Negative Binomial Regression for total number of events endpoints.
*Number of patients with event(s) for time to first event endpoints and number of events for total number of events endpoints.
†Number of patients with event(s) per 100 patient-years for time to first event endpoints and adjusted number of events per 100 patient-years (based on Negative Binomial Regression) for total number of event endpoints.

SUPPLEMENTARY FIGURE 3. MAJOR HEART FAILURE AND MORTALITY OUTCOMES (I)

Variable	Empagliflozin (n=3260)		Placebo (n=3262)		Hazard ratio or rate ratio‡	*p*-value
	N events*	Events/ 100 py†	N events*	Events/ 100 py†		
Time to first HHF or HF death	129	2.8	166	3.7	0.78 (0.62, 0.98)	0.031
Total number of HHF or HF death	168	2.9	236	4.2	0.69 (0.51, 0.93)	0.015
Time to first HHF or CV death	231	5.1	259	5.7	0.90 (0.75, 1.07)	0.228
Total number of HHF or CV death	280	6.3	338	7.2	0.88 (0.68, 1.14)	0.345

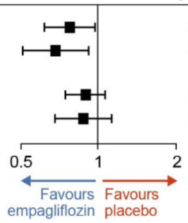

‡Hazard Ratio (95% confidence interval), p-value based on Cox proportional hazards model for time to first event endpoints, Event Rate Ratio (95% CI), based on Negative Binomial Regression for total number of events endpoints.
*Number of patients with event(s) for time to first event endpoints and number of events for total number of events endpoints.
†Number of patients with event(s) per 100 patient-years for time to first event endpoints and adjusted number of events per 100 patient-years (based on Negative Binomial Regression) for total number of event endpoints.

SUPPLEMENTARY FIGURE 4. MAJOR HEART FAILURE AND MORTALITY OUTCOMES (II)

(A) Time to first adverse event of heart failure and (B) Total number of heart failure hospitalizations or death due to heart failure

A

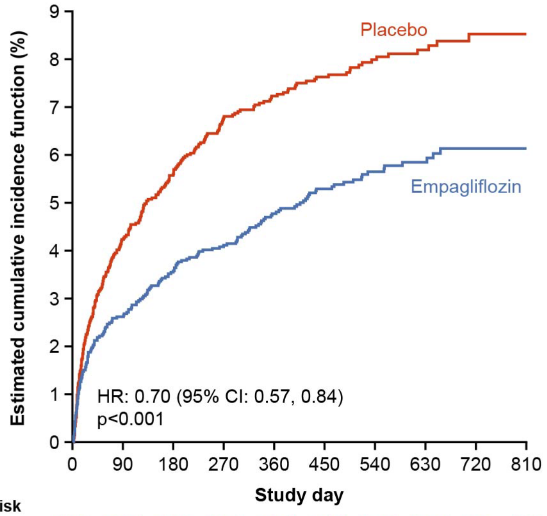

Patients at risk	0	90	180	270	360	450	540	630	720	810
Placebo	3262	3041	2975	2757	2421	2014	1512	1016	538	130
Empagliflozin	3260	3091	3030	2848	2497	2077	1539	1030	518	130

B

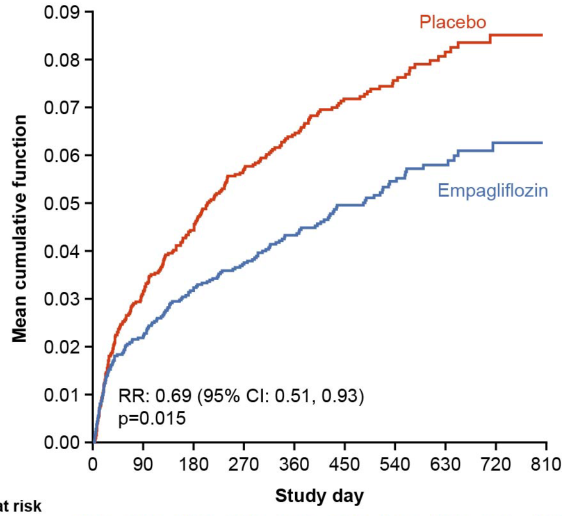

Patients at risk	0	90	180	270	360	450	540	630	720	810
Placebo	3262	3158	3125	2925	2579	2154	1617	1086	581	144
Empagliflozin	3260	3162	3124	2955	2600	2171	1616	1088	552	139

SUPPLEMENTARY FIGURE 5: ADVERSE EVENTS OF HEART FAILURE (I)
(A) TOTAL NUMBER OF ADVERSE EVENTS OF HEART FAILURE (B) TOTAL NUMBER OF ADVERSE EVENTS OF HEART FAILURE OR ALL-CAUSE MORTALITY (C) TIME TO FIRST ADVERSE EVENT OF HEART FAILURE REQUIRING/PROLONGING HOSPITALIZATION OR WITH FATAL OUTCOME
(D) TOTAL NUMBER OF ADVERSE EVENTS OF HEART FAILURE REQUIRING/PROLONGING HOSPITALIZATION OR WITH FATAL OUTCOME (E) TIME TO FIRST OUTPATIENT NON-FATAL ADVERSE EVENT OF HEART FAILURE
(F) TOTAL NUMBER OF OUTPATIENT NON-FATAL ADVERSE EVENTS OF HEART FAILURE

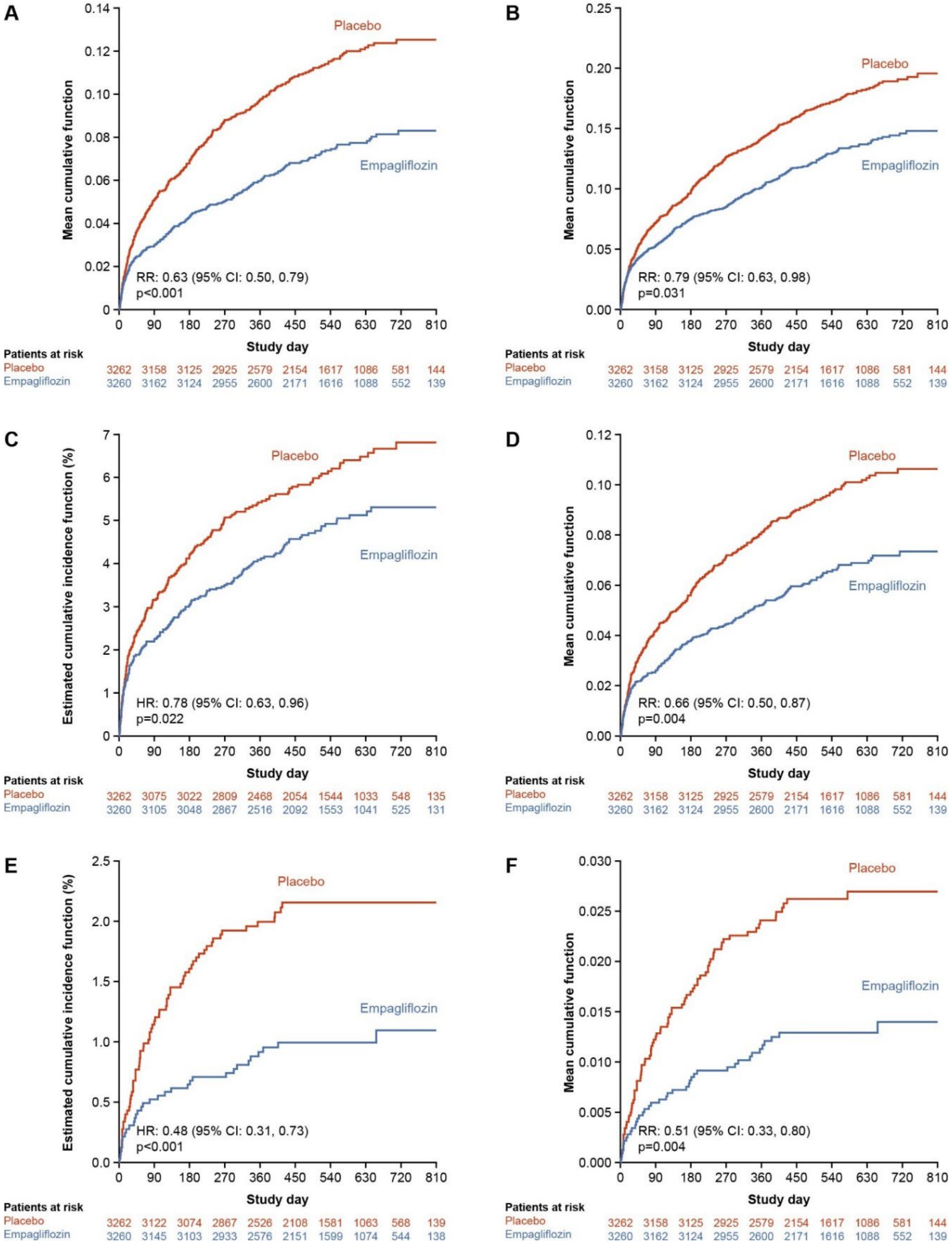

SUPPLEMENTARY FIGURE 6. ADVERSE EVENTS OF HEART FAILURE (II)

Variable	Empagliflozin (n=3260) N events*	Empagliflozin (n=3260) Events/ 100 py†	Placebo (n=3262) N events*	Placebo (n=3262) Events/ 100 py†	Hazard ratio or rate ratio‡	*p*-value
**Adverse events of heart failure**						
Time to first adverse event of HF	178	4.0	254	5.7	0.70 (0.57, 0.84)	<0.001
Total number of adverse events of HF	227	3.9	354	6.2	0.63 (0.50, 0.79)	<0.001
Time to first adverse event of HF requiring/prolonging hospitalization or with fatal outcome	154	3.4	197	4.4	0.78 (0.63, 0.96)	0.022
Total number of adverse events of HF requiring/prolonging hospitalization or with fatal outcome	200	3.5	297	5.4	0.66 (0.50, 0.87)	0.004
Time to first outpatient non-fatal adverse event of HF§	32	0.7	68	1.5	0.48 (0.31, 0.73)	<0.001
Total number of outpatient non-fatal adverse events of HF§	40	0.7	81	1.3	0.51 (0.33, 0.80)	0.004
Time to first adverse event of HF or all-cause mortality	313	6.9	377	8.5	0.83 (0.71, 0.96)	0.013
Total number of adverse events of HF or all-cause mortality	396	8.9	532	11.3	0.79 (0.63, 0.98)	0.031
Time to first adverse event of HF or CV death	279	6.2	340	7.7	0.82 (0.70, 0.96)	0.013
Total number of adverse events of HF or CV death	359	8.0	485	10.1	0.79 (0.63, 0.99)	0.043

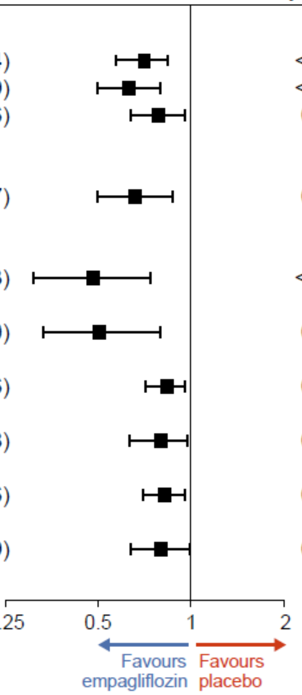

§Outpatient non-fatal adverse events of heart failure are defined as adverse events of heart failure that do not result in death nor require/prolong hospitalization.
‡Hazard Ratio (95% confidence interval), p-value based on Cox proportional hazards model for time to first event endpoints, Event Rate Ratio (95% CI), based on Negative Binomial Regression for total number of events endpoints.
*Number of patients with event(s) for time to first event endpoints and number of events for total number of events endpoints.
†Number of patients with event(s) per 100 patient-years for time to first event endpoints and adjusted number of events per 100 patient-years (based on Negative Binomial Regression) for total number of event endpoints.
